# Atypical cyclic di-AMP signaling is essential for *Porphyromonas gingivalis* growth and regulation of cell envelope homeostasis and virulence

**DOI:** 10.1038/s41522-022-00316-w

**Published:** 2022-07-06

**Authors:** M. Fata Moradali, Shirin Ghods, Heike Bähre, Richard J. Lamont, David A. Scott, Roland Seifert

**Affiliations:** 1 Department of Oral Immunology and Infectious Diseases, University of Louisville, School of Dentistry, Louisville, KY USA; 2 Research Core Unit Metabolomics, Hannover Medical School, Hanover, Germany

**Keywords:** Pathogens, Dentistry

## Abstract

Microbial pathogens employ signaling systems through cyclic (di-) nucleotide monophosphates serving as second messengers to increase fitness during pathogenesis. However, signaling schemes via second messengers in *Porphyromonas gingivalis*, a key Gram-negative anaerobic oral pathogen, remain unknown. Here, we report that among various ubiquitous second messengers, *P. gingivalis* strains predominantly synthesize bis-(3′,5′)-cyclic di-adenosine monophosphate (c-di-AMP), which is essential for their growth and survival. Our findings demonstrate an unusual regulation of c-di-AMP synthesis in *P. gingivalis*. *P. gingivalis* c-di-AMP phosphodiesterase (PDE) gene (*pde*_*pg*_) positively regulates c-di-AMP synthesis and impedes a decrease in c-di-AMP concentration despite encoding conserved amino acid motifs for phosphodiesterase activity. Instead, the predicted regulator gene *cdaR*, unrelated to the c-di-AMP PDE genes, serves as a potent negative regulator of c-di-AMP synthesis in this anaerobe. Further, our findings reveal that *pde*_*pg*_ and *cdaR* are required to regulate the incorporation of ATP into c-di-AMP upon pyruvate utilization, leading to enhanced biofilm formation. We show that shifts in c-di-AMP signaling change the integrity and homeostasis of cell envelope, importantly, the structure and immunoreactivity of the lipopolysaccharide layer. Additionally, microbe–microbe interactions and the virulence potential of *P. gingivalis* were modulated by c-di-AMP. These studies provide the first glimpse into the scheme of second messenger signaling in *P. gingivalis* and perhaps other Bacteroidetes. Further, our findings indicate that c-di-AMP signaling promotes the fitness of the residents of the oral cavity and the development of a pathogenic community.

## Introduction

Periodontitis is a highly prevalent and destructive infectious, inflammatory disease of the tissues supporting the teeth that is induced by a synergistic community of virulent subgingival bacteria^1,2^. Among recognized pathogens, the Gram-negative anaerobe *Porphyromonas gingivalis* has been strongly implicated in the onset and development of periodontitis and several systemic diseases in adults upon producing an array of virulence factors, orchestrating dysbiotic inflammation, and disrupting host–microbial homeostasis even at low abundance^2–12^. Yet, how *P. gingivalis* can persist in the subgingival niche, induce ecological changes, and promote polymicrobial infection remain obscure. *P. gingivalis* has long been described as a highly proteolytic and asaccharolytic pathogen. It utilizes the type IX secretion system (T9SS) and T9SS cargo proteins, such as potent proteases known as gingipains, to provide proteinaceous nutrients while enabling the manipulation of immune components^4,13–15^. Our recent work revealed that the metabolic plasticity of *P. gingivalis* allows the consumption of exogenous pyruvate as an important source of carbon and energy^16,17^. Utilization of pyruvate significantly rewires the central metabolism of *P. gingivalis* toward de novo biosynthesis of phosphorylated and non-phosphorylated carbohydrates, promotes the expression of fimbrial adhesins, and subsequently enhances biofilm formation, co-colonization of *P. gingivalis* and other accessory species, and invasion^16,17^. Metabolomics data^16^ suggested that cyclic (di-) nucleotide monophosphates may broadcast metabolic signals in *P. gingivalis*. Still, the scheme of signaling through second messengers and their biological roles in this anaerobe and other Bacteroidetes remain largely unknown. Signaling systems via cyclic (di-) nucleotide monophosphate second messengers have been long described as fundamental regulatory mechanisms by which bacterial pathogens can promptly respond to specific stimuli and control appropriate biological outputs and pathoadaptive mechanisms, including phenotypic changes, the motility-sessility switch, host colonization, biofilm formation, virulence, stress tolerance, and antibiotic resistance^18–21^. Paradoxically, Chaudhuri et al.^22^ reported the presence of bis-(3′,5′)-cyclic di-guanosine monophosphate (c-di-GMP) signaling in *P. gingivalis* while pioneering works indicated that neither c-di-GMP metabolizing enzymes nor c-di-GMP receptors exist in Bacteroidetes^23,24^. Such inconsistent findings justify further investigation of signaling systems in *P. gingivalis* and other periodontopathogens.

In this study, we sought to reveal the scheme of nucleotide-based signaling in *P. gingivalis* strains using direct analytical evidence and approaches, and to determine biological significance during pathogenesis. Our data demonstrate that bis-(3′,5′)-cyclic di-adenosine monophosphate (c-di-AMP) is the primary and essential signaling system in *P. gingivalis*, which controls cell growth, cell wall integrity, biofilm formation, and virulence. Since c-di-AMP signaling does not exist in mammals, it is potentially a novel druggable target for controlling pathogens belonging to the phylum Bacteroidetes.

## Results

### Analysis of nucleotide-based signaling molecules in *P. gingivalis*

**Fig. 1:**
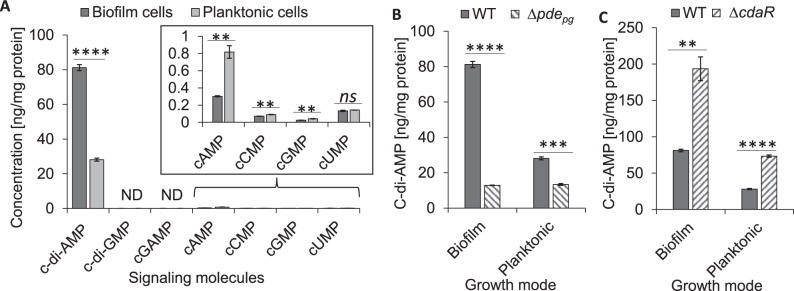
Comprehensive analysis of cyclic mono- and di-nucleotide second messengers in *P. gingivalis* 381. **A** Cellular concentration of ubiquitous bacterial second messengers in the cells of *P. gingivalis* in biofilm or planktonic modes of growth. Mononucleotide second messengers are in the low-nanogram range (inside box). **B** c-di-AMP in *P. gingivalis* ∆*pde*_*pg*_. **C** c-di-AMP in *P. gingivalis* ∆*cdaR*. Graphs represent the mean ± SE (three biological replicates) of nucleotide second messengers which were analyzed with a student’s *t* test (**P < 0.01; ***P < 0.001; ****P < 0.0001; ns not significant). Scatter plots in Supplementary Fig. 9 display the data distribution. Standard curves are presented in Supplementary Fig. 10. ND not detected, WT wild type.

It is noteworthy to mention that genetic and phenotypic variability, e.g., fimbriation vs. encapsulation, exists among clinically prevalent strains of *P. gingivalis*, which significantly impacts host–pathogen interactions and virulence potential^25,26^. Here we sought to provide a first glimpse into signaling schemes of two phenotypically different strains 381 (hyperfimbriated/non-encapsulated) and W50 (encapsulated/fimbriated), with emphasis on strain 381. Strain 381 is a robust biofilm former exhibiting high invasion efficiency, which induces proinflammatory cytokines and periodontal bone loss^27,28^. We analyzed ubiquitous bacterial cyclic nucleotide monophosphates (cNMPs) and cyclic di-nucleotide monophosphates (c-di-NMPs) in metabolic extracts of biofilm- and planktonic-grown cells of strain 381. We found that c-di-AMP is the most abundant signaling molecule in planktonic and biofilm cells of *P. gingivalis* (Fig. 1A). Other c-di-NMPs, i.e., c-di-GMP and 2′3′-cyclic GMP-AMP (cGAMP) were not detected. Among detected cNMPs, the cellular concentration of cAMP was less than 0.9 ng per mg protein in strain 381. In contrast, we quantified cCMP, cGMP, and cUMP concentrations in the low-nanogram range (<0.15 ng per mg protein). Planktonic cells of strain 381 displayed higher concentrations of cNMPs than biofilm cells; the cellular concentration of cAMP in planktonic cells was almost 2.7 times higher than biofilm cells (Fig. 1A). Similar to strain 381, the cellular concentration of c-di-AMP in strain W50 (a weak biofilm former) was the greatest among detected molecules. However, it was almost 7.8 times less than strain 381 (Supplemental Fig. 1A). Similarly, the concentration of cNMPs in strain W50 was in the low-nanogram range, and the cAMP concentration in strain W50 was about two times higher than strain 381 (Supplemental Fig. 1B), indicating c-di-AMP and cAMP may distinctively associate with specific phenotypes and strains in this species. Overall, our data demonstrate that *P. gingivalis* strains do not possess c-di-GMP signaling; instead, they predominantly harness c-di-AMP signaling, which positively correlates with the potential for biofilm formation. Supplementary Table 1 represents the mean values of cNMPs, c-di-NMPs, and other molecules in pmol/mg protein.

### C-di-AMP turnover in *P. gingivalis* in response to biologically relevant stimuli

Given that c-di-AMP was the predominant second messenger in *P. gingivalis* strains, we sought to investigate the molecular mechanism of c-di-AMP signaling, as determined by highly conserved c-di-AMP-metabolizing enzymes in prokaryotes, i.e., di-adenylate cyclases (DACs) and the DHH/DHHA1- or PgpH/HD (or His-Asp)-type domain-containing phosphodiesterases (PDEs)^29,30^. In general, these enzymes collectively control the delicate equilibrium between the synthesis of c-di-AMP from two molecules of ATP or its hydrolysis into 5′-phosphadenylyl-adenosine (pApA) or two molecules of adenosine monophosphate (AMP) in response to perceived stimuli. Subsequently, a specific cellular concentration of c-di-AMP must engage specific receptors/effectors to control desired biological outputs^20,21,31,32^. Using NCBI BLAST and the Phyre2 Protein Fold Recognition Server^33^, we found that the products of two nearby genes PGN_0523 and PGN_0521 have 36% and 38% structural homology with *Listeria monocytogenes* DAC and PgpH/HD-type PDE enzymes, respectively. We did not find a DHH-DHHA1 domain-containing c-di-AMP PDEs in *P. gingivalis*. In addition, multiple sequence alignments of these proteins predicted highly conserved motifs for mediating c-di-AMP binding and fulfilling synthesis or degradation processes (Supplemental Fig. 2). Hence, we posited that deletion of PGN_0523 (∆*dac*_*pg*_) or PGN_0521 (∆*pde*_*pg*_) would change the cellular concentration of c-di-AMP significantly. Our attempt to generate a *dac*_*pg*_ deletion mutant was unsuccessful, indicating that *dac*_*pg*_ is essential for *P. gingivalis* growth. This finding is consistent with previous reports demonstrating that c-di-AMP synthases are necessary for the growth of several bacterial species^34^ and *dac*_*pg*_ was identified among inherently essential genes in *P. gingivalis* based on transposon sequencing libraries^35–37^. Strikingly, deletion of the *pde*_*pg*_ gene, which is typically expected to elevate c-di-AMP concentration, significantly reduced it by 2.1- and 6.3-fold in planktonic- and biofilm-grown cells, respectively, when compared to the wild type (Fig. 1B and Supplemental Fig. 3A). While in *trans* complementation of the *pde*_*pg*_ mutant was not achievable, in *cis* complementation with *pde*_*pg*_ yielded increased c-di-AMP concentration to the same concentration of wild-type c-di-AMP (Supplemental Fig. 3B). To the best of our knowledge, this is the first c-di-AMP PDE enzyme that positively regulates the cellular concentrations of c-di-AMP among bacteria.

Upon further investigation to find a gene candidate acting as a negative regulator of c-di-AMP turnover, the STRING database^38^ suggested PGN_1486 encoding a putative YbbR- or CdaR-like protein may be functionally associated with the c-di-AMP signaling network in *P. gingivalis*. Interestingly, deletion of PGN_1486 (∆*cdaR*) significantly increased c-di-AMP concentrations by about 2.6- and 2.4-fold, in planktonic and biofilm cells, respectively (Fig. 1C and Supplemental Fig. 3A). *cis* complementation of ∆*cdaR* with *cdaR* reduced c-di-AMP concentrations to the same concentration as wild-type c-di-AMP (Supplemental Fig. 3B), which rules out polar effects on nearby genes.

A comparison of the cellular concentration of cNMPs in the mutants showed that they do not significantly change between ∆*pde*_*pg*_ and ∆*cdaR* in biofilm mode, although both mutants contained much less cAMP concentration than the wild type (Supplemental Fig. 3C). However, in planktonic mode, the deletion of *cdaR* almost doubled the cellular concentrations of cCMP, cGMP, and cUMP and impeded the reduction of cAMP (Supplemental Fig. 3D). Overall, these data suggest crosstalk between the c-di-AMP signaling pathway and other signaling systems in *P. gingivalis* only in the planktonic mode of growth.

**Fig. 2:**
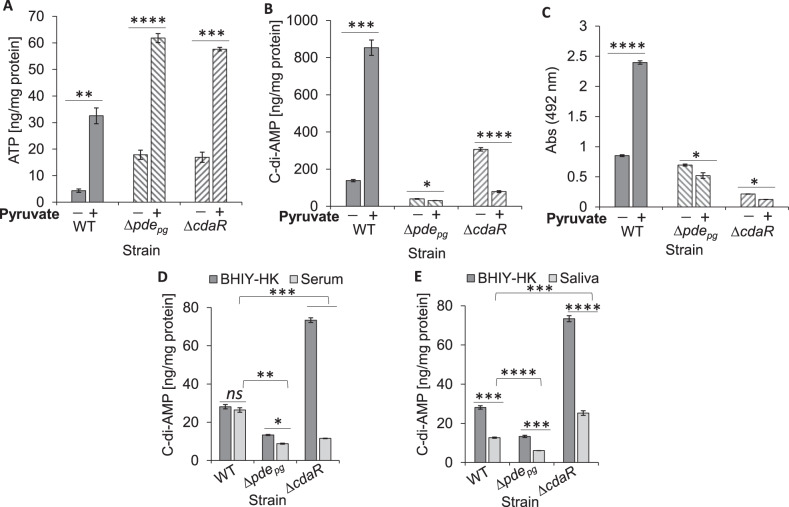
Both *pde*_*pg*_ and *cdaR* genes are required for the synthesis of c-di-AMP and biofilm formation while they regulate differentially the cellular level of c-di-AMP in *P. gingivalis* in response to biologically relevant stimuli. **A**, **B** Cellular levels of ATP and c-di-AMP in biofilm cells in response to pyruvate availability. **C** Monospecies biofilm formation with WT, ∆*pde*_*pg*_, and ∆*cdaR*; in the presence or absence of pyruvate. **D**, **E** Differential regulation of c-di-AMP levels in the WT, ∆*pde*_*pg*_, and ∆*cdaR* mutants in response to 10% human serum (**D**) or 10% saliva (**E**). Graphs represent the mean ± SE (three biological replicates) of c-di-AMP concentrations which were analyzed with a Student’s *t* test (*P < 0.05; **P < 0.01; ***P < 0.001; ****P < 0.0001; ns not significant). Scatter plots in Supplementary Fig. 9 display the data distribution. Standard curves are presented in Supplementary Fig. 10. WT wild-type.

In order to provide more insights into the mechanism of c-di-AMP turnover, the products of c-di-AMP degradation, i.e., pApA or AMP, were quantified in the wild-type and mutants. Similar to c-di-AMP quantification data, ∆*pde*_*pg*_ and ∆*cdaR* cells contained lower and higher pApA and/or AMP concentrations respectively, compared to wild type (Supplemental Fig. 3E, F). However, alterations in pApA or AMP concentrations in the mutants were disproportionate to quantified c-di-AMP concentrations, indicating that c-di-AMP degradation may not typically occur in *P. gingivalis*. Therefore, we hypothesize that the availability of ATP may regulate c-di-AMP signaling at the synthesis level in this organism. Quantification of cellular ATP showed that ∆*pde*_*pg*_ and ∆*cdaR* mutants significantly accumulate ATP three to four times more than the wild type (Supplemental Fig. 4). These data suggest that PDE_pg_ and CdaR are critical for determining c-di-AMP equilibrium through controlling the synthesis process rather than hydrolysis. As a proof of concept, we used pyruvate as a source of carbon and energy for *P. gingivalis*^16,17^ to provide a permissive condition for in situ provision of ATP followed by simultaneous quantification of ATP and c-di-AMP. Intriguingly, pairwise comparisons showed that although pyruvate utilization increased ATP concentrations significantly in ∆*pde*_*pg*_ and ∆*cdaR* mutants compared to the wild-type (Fig. 2A), it elevated the c-di-AMP concentration significantly only in the wild-type and decreased it in the mutants (Fig. 2B). Consistent with these findings, pyruvate addition greatly enhanced biofilm formation of the wild-type while reducing the mutants’ ability to form a biofilm (Fig. 2C). These data indicate that coexistence of PDE_pg_ and CdaR is required to regulate the incorporation of ATP into c-di-AMP synthesis in *P. gingivalis* and regulate biofilm formation through a yet to be determined mechanism.

To assess the response of c-di-AMP signaling to biologically relevant stimuli, we applied human serum or saliva as primary constituents of subgingival and supragingival environments, respectively. To this end, Brain Heart Infusion (supplemented with yeast extract, hemin, and menadione (BHIYHK))-grown cells in exponential phase (OD_600_: 0.65; 5 × 10^8^ CFU) were incubated in 10% human serum or saliva for 30 min followed by extraction and quantification of molecules. The cellular concentration of c-di-AMP remained unchanged in the wild type in response to serum but was reduced in both mutants (Fig. 2D). Saliva reduced the concentration of c-di-AMP in the wild-type and mutants (Fig. 2E). Overall, these data suggest that c-di-AMP signaling is regulated differently in response to different stimuli in the oral cavity, and that both PDE_pg_ and CdaR are necessary for maintaining optimal concentrations of c-di-AMP, particularly in the subgingival environment. Supplementary Table 1 represents the mean values of cNMPs, c-di-NMPs, and other molecules in pmol/mg protein.

### Cell growth and biofilm formation are regulated by c-di-AMP turnover in *P. gingivalis*

We first assessed the colony morphology and growth rate of the ∆*pde*_*pg*_ and ∆*cdaR* mutants. In general, strain 381 displays a smooth appearance on BHIYHK plates but colonies of ∆*pde*_*pg*_ displayed a glossier appearance, while ∆*cdaR* appeared as rough colonies (Supplemental Fig. 5A). Given that *P. gingivalis* intrinsically grows slowly but tends to auto-aggregate progressively in bovine serum albumin (BSA)-only medium over short periods^16,39^, we used this condition to assess cell surface properties of ∆*pde*_*pg*_ and ∆*cdaR* mutants in static liquid cultures. ∆*cdaR* cells demonstrated strong cohesiveness and auto-aggregation, whereas ∆*pde*_*pg*_ cells showed less auto-aggregation compared to the parental strain (Supplemental Fig. 5B). Both mutants were unaffected in their ability to form black-pigmented colonies on blood agar plates due to heme accumulation on the cell surface. However, the mutants did not display hemolytic activity on blood agar (Supplemental Fig. 5C). These findings suggest that c-di-AMP signaling in *P. gingivalis* can impact cell surface properties.

**Fig. 3:**
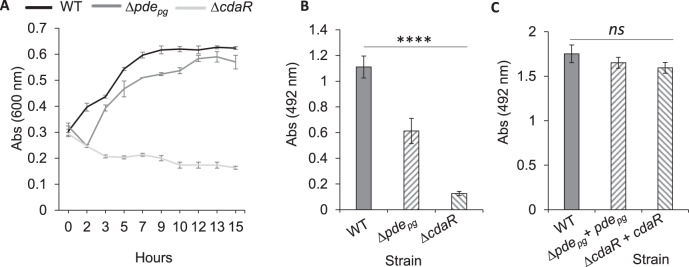
Comparison of planktonic growth rate and biofilm formation. **A** Graph shows that ∆*pde*_*pg*_ displays a lower rate of planktonic growth than the wild-type and ∆*cdaR* displays a significant growth defect. Cells were cultivated in the CDM-HSA-HK medium. **B** Biofilm assay using 96-well plates shows that both mutants produce less biofilms than WT in the CDM-HSA-HK medium. However, complementation of the mutants with relevant genes restored biofilm formation to the level of the wild type (**C**). Graphs represent mean biomass of biofilms ± SE (three biological replicates) at 48 h, as determined by safranin staining, which were analyzed using ANOVA test (Shapiro–Wilk test: *P* > 0.05). Asterisks indicate pairs of significantly different values (post hoc Tukey’s HSD test: ****P < 0.0001; ns not significant).

**Fig. 4:**
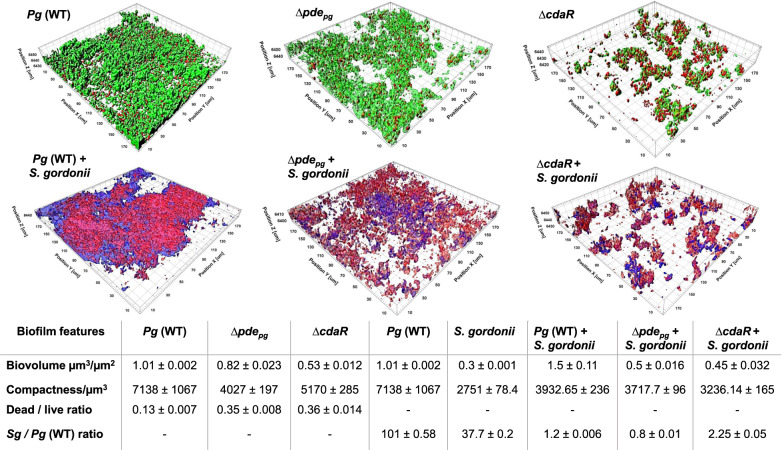
Effects of defective regulation of c-di-AMP levels on biofilm formation of *P. gingivalis* WT, ∆*pde*_*pg*_, and ∆*cdaR* and its colocalization with *S. gordonii*. CLSM images of mono- and dual-species biofilms were analyzed by IMARIS image analysis software. The resultant biofilm parameters are summarized in the table. The cell community dimensions are provided as µm^3^/µm^−2^ and the values of three biological replicates were calculated per unit that represent mean biovolume/unit ± SE, compactness/unit ± SE, dead/live ratio, and *S. gordonii*/*P. gingivalis* ratio, as determined by appropriate staining methods. *Sg* *S. gordonii*, *Pg* *P. gingivalis*, WT wild-type.

In chronic periodontal infection, *P. gingivalis* interacts with a complex microbial community in the subgingival crevice, between the tooth surface and the adjacent epithelium where a serum exudate (known as gingival crevicular fluid) constitutes the primary nutritional flow^5,40^. Our previous findings demonstrated that *P. gingivalis* could efficiently utilize serum components which play an ancillary role in developing polymicrobial biofilms upon establishing physical and cross-feeding networks with accessory species^16^. Here, we applied similar experimental conditions, i.e., the use of human serum albumin (HSA)- or human serum-based media that offer biologically relevant model systems for studying proteolytic activity, biofilm formation, and in situ interactions between different oral species^16,17,39^. Analysis of planktonic growth rate showed that ∆*pde*_*pg*_, and to a much greater degree, ∆*cdaR* displayed a significant growth defect in chemically defined medium supplemented with human serum albumin (CDM-HSA-HK) (Fig. 3A). In monospecies biofilm accumulation in microtiter plates, the ∆*pde*_*pg*_ and ∆*cdaR* mutants formed almost two- and six-fold less biofilm than the wild type, while the biofilm phenotype was restored to parental concentrations in *cis*-complemented strains (Fig. 3B, C). Consistent with these data, quantitative image analysis of biofilms grown in the CDM-Serum-HK medium revealed that ∆*pde*_*pg*_ forms a less developed biofilm with a lower biovolume value compared to the wild type. The ∆*cdaR* mutant displayed a greater reduction in the ability to form biofilms. Further, the biofilms of both mutants contained higher numbers of dead cells and displayed lower compactness values than the wild-type (Fig. 4).

Given that our prior findings demonstrated that among different oral species, only *P. gingivalis* could efficiently exploit serum components for the development of polymicrobial biofilm with *Streptococcus gordonii*^16^, we hypothesized that the ∆*pde*_*pg*_ and ∆*cdaR* mutants may display different capabilities of co-colonization with *S. gordonii*. Quantitative image analysis revealed an efficient co-colonization of *P. gingivalis* and *S. gordonii* to form dual-species communities with both species almost equally present (Fig. 4 and Supplemental Fig. 6). However, deletion of *pde*_*pg*_ or *cdaR* significantly changed the interaction of *P. gingivalis* with *S. gordonii*, resulting in a threefold or more decrease of biofilm volume and compactness. Moreover, the *S. gordonii* population was almost double the population of the ∆*cdaR* mutant in their mixed biofilm (Fig. 4). Overall, these data indicate that changes in c-di-AMP concentrations significantly alter biofilm features and microbial interactions between *P. gingivalis* and accessory species.

### C-di-AMP signaling controls cell wall homeostasis and stress tolerance

**Fig. 5:**
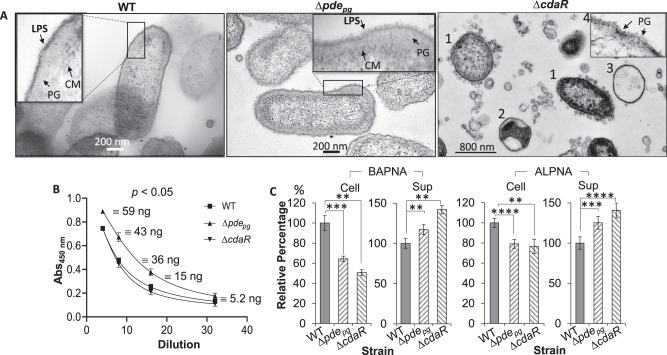
Effects of defective regulation of the cellular c-di-AMP level on *P. gingivalis* cell envelope, the immunoreactivity of LPS, and gingipain activities. **A** Cells were stained with osmium tetroxide and uranyl acetate and imaged by transmission electron microscopy. Cells of ∆*cdaR* mutant displayed a significant shape and cell envelope heterogeneity represented by rod- and round-shaped cells with fully or partially intact cell envelopes that are overloaded with OMVs varying in shape and size (1); cells with an intact monolayer of membrane encompassing agglomerated cytoplasmic materials (2) or entirely void round-shaped structures with an intact monolayer of the membrane (3), cells displaying bare peptidoglycan layers (4). **B** ELISA assays of cell lysates using anti-*P. gingivalis* LPS monoclonal antibody. The graph represents the mean ± SE of the immunoreactivity of cell lysates (three biological replicates). The standard curve is presented in Supplementary Fig. 10. **C** Gingipain-dependent proteolytic activities of *P. gingivalis* strains cells. Graphs represent the mean ± SE (three biological replicates) of the activity of arginine (BAPNA) and lysine (ALPNA) gingipains which were analyzed with a Student’s *t* test (**P < 0.01; ***P < 0.001; ****P < 0.0001). Scatter plots in Supplementary Fig. 9 display the data distribution. Sup cell-free supernatant, BAPNA N-α-benzoyl-l-arginine-p-nitroanilide, ALPNA N-α-acetyl-l-lysine-p-nitroanilide.

To examine directly if c-di-AMP signaling impacts cell surface and membrane structures, we performed transmission electron microscopy (TEM) on biofilm cells of the wild type and mutants. TEM images revealed a network of macromolecules extending much further from the less uniform outer leaflet of the outer membrane (OM) of ∆*pde*_*pg*_ cells compared to the wild type (Fig. 5A and Supplemental Fig. 7A, B). We posited that this network of macromolecules comprises lipopolysaccharides (LPSs) since the sample preparation procedure predominantly stains phosphoryl and carboxyl groups of lipids and lipopolysaccharides, the outer face of the OM is primarily composed of LPSs, and strain 381 does not produce capsular polysaccharides^41–43^. Intriguingly, the cells of ∆*cdaR* mutant displayed a significant heterogeneity in cell shape and cell envelope as follows: (1) rod- and round-shaped cells with fully or partially intact envelopes that are overloaded with outer membrane vesicles (OMVs) varying in shape and size; (2) round cells with an intact monolayer of membrane encompassing agglomerated cytoplasmic materials with void spaces in the cells; (3) entirely void round-shaped structures with an intact monolayer of membrane; and (4) cells without recognizable OM layers which display a bare peptidoglycan layer (Fig. 5A and Supplemental Fig. 7C, D). Such heterogeneity was also distinguishable in confocal laser microscopy images (Supplemental Fig. 7E), supporting our notion that observed structural heterogeneity in cells and their envelope was caused by the deletion of the *cdaR* gene, rather than the sample preparation procedure.

To supplement the TEM-based observations, we performed an ELISA assay using anti-*P. gingivalis* LPS monoclonal antibody on cell lysates of planktonic cells from the exponential phase of growth. ∆*pde*_*pg*_ cells exhibit higher immunoreactivity to the antibody than the wild type, while ∆*cdaR* cells show slightly less (*P* < 0.05) (Fig. 5B).

In general, changes in LPS composition affect cell wall homeostasis, hence the sensitivity of Gram-negative bacteria to antibiotics and environmental stresses^44–46^. To assess if c-di-AMP-dependent changes in the cell envelope impact *P. gingivalis* susceptibility to stresses, we measured the minimum inhibitory concentration (MIC) of amoxicillin and sensitivity to sodium dodecyl sulfate (SDS) for each strain. The MIC test showed that deletion of *pde*_*pg*_ or *cdaR* significantly increased *P. gingivalis* susceptibility to amoxicillin by about fivefold and tenfold, respectively, compared to the wild-type (Supplemental Fig. 8A). Similarly, the ∆*pde*_*pg*_ and ∆*cdaR* mutants displayed greater sensitivity to SDS and were completely lysed in the presence of 0.001% SDS (Supplemental Fig. 8B). Overall, these data indicate that c-di-AMP signaling regulates the composition of LPS in *P. gingivalis* directly or indirectly and plays a significant role in cell envelope homeostasis and protection against stresses.

### C-di-AMP signaling controls the virulence potential of *P. gingivalis*

Since A-LPS variant of *P. gingivalis* is employed for post-translational modification of gingipains and anchorage on the cell surface^47,48^, we hypothesized that such heterogeneity in LPS structure may impact the association of gingipains on the surface of the mutants. A gingipain assay^49^ showed that the cell-free supernatants of both mutants show more gingipain activity, and cell surfaces have less activity, compared to the wild-type (Fig. 5C).

**Fig. 6:**
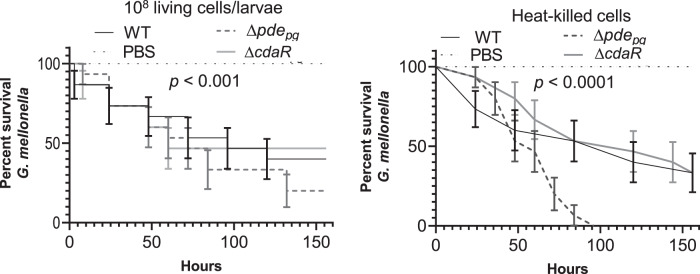
Survival of *G. mellonella* larvae injected with ∼10^8^ CFU/ml of *P. gingivalis* WT, ∆*pde*_*pg*,_ and ∆*cdaR* mutants. The control group was injected with sterile PBS. Survival data were plotted using the Log-rank (Mantel–Cox) test. Graph shows the average of two biological replicates and the standard error (*n* = 15 larvae per group).

To assess the virulence potential of the mutants, we applied the *Galleria mellonella* infection model, an increasingly popular model for determining the virulence of bacterial pathogens, including periodontopathogens, because of similarities with the mammalian innate immune system^50–52^. Living cells of the ∆*pde*_*pg*_ mutant exhibited higher virulence with only 20% larval survival after 156 h of infection (Fig. 6). On the other hand, living cells of the ∆*cdaR* mutant were slightly less virulent than the wild type. Further, the release of metabolites and lipid-based contents by heat treatment increased the toxicity of the ∆*pde*_*pg*_ mutant (0% larval survival after 100 h of infection) (Fig. 6). Heat-killed cells of the ∆*cdaR* mutant showed less toxicity than that of the wild-type within 60 h of the assay, but both resulted in 33% larval survival after 156 h (Fig. 6). Overall, our data indicate that the c-di-AMP signaling pathway is associated with the virulence potential of *P. gingivalis*, and *pde*_*pg*_ and *cdaR* act oppositely in the determination of c-di-AMP concentration in the cells and virulence potential.

## Discussion

The results presented in this study reveal the schemes of nucleotide-based second messenger signaling in *P. gingivalis*. This anaerobe is a key player in the etiology of periodontitis and may be an important paradigm for signaling mechanisms via nucleotide-based second messengers in Bacteroidetes. We found that, among ubiquitous cNMPs and c-di-NMPs, *P. gingivalis* strains predominantly synthesize c-di-AMP, which is essential for their growth and survival. Consistent with previous reports indicating that the c-di-GMP signaling is absent in Bacteroidetes^23,24^, we could not detect c-di-GMP or cGAMP in *P. gingivalis* strains. To date, the essential role of c-di-AMP signaling for bacterial growth, and their role in controlling a wide range of cellular and biological processes, including biofilm formation, central metabolism, cell wall homeostasis, and osmoregulation, have been addressed primarily in Gram-positive species^20,34,53–61^ and our understanding of its biological significance in the pathogenesis of Gram-negative pathogens remain largely limited. Emerging data indicate that the c-di-AMP signaling mechanism follows the same general principle as for other second messengers in which cognate metabolizing enzymes control the equilibrium between synthesis and degradation of c-di-AMP molecules, which are necessary for engaging specific protein or riboswitch receptors to act as direct effectors for regulating desired biological outputs^62,63^. Here, we show that as opposed to this general principle, as determined by DAC and the DHH/DHHA1- or PgpH/HD domain-containing PDE enzymes, respectively, the concentration of c-di-AMP is positively regulated by *pde*_*pg*_ in *P. gingivalis*. In addition, low cellular concentrations of pApA or AMP (c-di-AMP degradation products) and their disproportionate relationship to c-di-AMP concentrations suggest that, at least under our experimental condition, PDE_pg_ may not act as a c-di-AMP phosphodiesterase in *P. gingivalis* despite displaying conserved amino acid motifs with functionally related proteins. Instead, PDE_pg_ impedes a decrease in c-di-AMP concentrations. However, its exact function(s) or the necessity of specific stimuli to induce a phosphodiesterase activity or the existence of an alternative mechanism or gene for c-di-AMP degradation in this species awaits further investigation. Intriguingly, we found that the *cdaR* gene, unrelated to the c-di-AMP PDE enzymes, serves as a potent negative regulator of c-di-AMP synthesis. In general, Ybbr domain-containing proteins (also known as CdaA regulatory protein CdaR in Gram-positive species) have been characterized as membrane proteins with sensory domains for an unknown signal^59,64^. It has been noted that *cdaR* and *dac* genes are often clustered together in one operon, and their products interact to regulate the c-di-AMP synthesis, and hence downstream cellular pathways^58,65^. However, different CdaR proteins have been found to act oppositely on the c-di-AMP synthesis in different species, such as *Bacillus subtilis* and *L. monocytogenes*^59,66^. In *P. gingivalis*, these two genes are located in two different genomic regions. Our findings indicate that simultaneous involvement of *dac*_*pg*_, *pde*_*pg*_, and *cdaR* is not only necessary for regulation of c-di-AMP synthesis and cell homeostasis but also essential for growth, biofilm formation, and fitness in different microenvironments. Interestingly, the high cellular concentration of c-di-AMP, achieved upon pyruvate supplementation, did not perturb wild-type cell hemostasis, but instead enhanced cell proliferation and biofilm biomass. Hence, we can conclude that the proposed toxicity of excess c-di-AMP in bacteria may not be the case in *P. gingivalis*, likely because the counterparts DAC_pg_, PDE_pg_, and CdaR may exert other activities that mitigate directly or indirectly such an adverse effect. Previous studies have addressed the necessity of c-di-AMP signaling for cell wall synthesis and metabolic homeostasis in the Gram-positive Firmicutes to cope with stresses and damages^20,31,55,67^, and our data indicate that c-di-AMP signaling and/or the activities of cognate protein counterparts can regulate the structure of LPS and cell envelope integrity of Gram-negative species, hence susceptibility to membrane-targeting antimicrobials. Notably, the absence of *cdaR* significantly dysregulates the integrity of the cell envelope and cellular homeostasis, leading to a membrane with numerous OMV-like and unusual membranous structures. *P. gingivalis* can produce a copious amount of OMVs loaded with various virulence factors, including LPS and gingipains, for dissemination and distal impacts^39,68,69^. However, the mechanistic basis of OMV biogenesis remains unknown. Our findings suggest CdaR is a strong candidate in the regulation of OMV biogenesis, and further investigation is ongoing to understand its exact function.

It is plausible that *P. gingivalis* has evolved c-di-AMP signaling for appropriate responses and enhanced fitness in the surrounding environment, which is a complex polymicrobial community with serum (subgingival) or saliva (supragingival) fluid phases. We found that c-di-AMP signaling is differentially regulated in response to human saliva and serum, indicating distinct functions in different microenvironments. Previous studies, including ours, demonstrated co-colonization and physical interaction of *P. gingivalis* with other potential pathogens, as well as the ancillary role of *P. gingivalis* in the establishment of metabolic crosstalk within the polymicrobial community upon the utilization of human serum components^2,16,70,71^. Further, a mixed community of *P. gingivalis* and *S. gordonii* is more pathogenic than *P. gingivalis* alone^72,73^. Our findings demonstrate that a c-di-AMP signaling pathway is required for the optimal interaction of *P. gingivalis* and *S. gordonii*. Our work also provided the first glimpse into the c-di-AMP-dependent variation of LPS that subsequently affects the association of T9SS cargo proteins such as gingipains to the cell surface. Both LPS and gingipains are notable virulence factors playing prominent roles in the pathological outcome of periodontitis by being involved in the stimulation of proinflammatory responses, tissue destruction, host-defense perturbation, and bone resorption^74–76^. Previous studies have addressed LPS heterogeneity in *P. gingivalis* in response to certain stimuli^77,78^ but the mechanistic basis of the shifts in the LPS profile remains obscure. Here, we revealed that *P. gingivalis* LPS profile and virulence potential change concomitant with c-di-AMP signaling alterations. The control of virulence potential via shifting the structure and immunostimulatory properties of LPS is an essential aspect of bacterial pathoadaptation by which pathogens can disguise themselves from host detection, regulate virulence and immunostimulatory effects to promote persistence in the host or protect themselves from host-defense mechanisms, antimicrobials, and environmental stresses^79–81^. Hence, we consider the c-di-AMP-dependent LPS heterogeneity in *P. gingivalis* as a novel mechanism for regulating pathogenesis and virulence. This notion is further supported by our findings that *P. gingivalis* displays a higher level of LPS immunoreactivity, as well as virulence and toxicity in the absence of *pde*_*pg*_ in our infection model. However, further investigations are required to reveal the mechanistic basis of the regulation of cellular pathways by c-di-AMP signaling and their biological importance in the onset and development of periodontitis by using different infection models.

Overall, to our knowledge, the unusual nature of c-di-AMP turnover in *P. gingivalis* and its involvement in regulating LPS profile and cell envelope integrity is unique to this species. Since the c-di-AMP signaling and cognate regulatory proteins are absent in mammals, they can be attractive antimicrobial drug targets for treating chronic infections such as periodontitis. However, further understanding of the role of c-di-AMP signaling in microbial dysbiosis, host–pathogen interaction, and periodontal inflammation is required to develop more precise and informed therapeutic interventions.

## Methods

### Ethics statement

Saliva collection was approved by the University of Louisville IRB, Protocol # 21.0925 and categorized as a prospective collection of biological specimens for research purposes by noninvasive means. Written informed consent was obtained from all participants.

### Bacterial strains, growth conditions, and chemicals

Bacteria used in this study were *P. gingivalis* strains 381 (with isogenic mutants described below) and W50, *Escherichia coli* strain DH5-α (New England BioLabs GmbH), and *Streptococcus gordonii* DL-1. Bacto™ Trypticase Soy Broth (BD Biosciences) supplemented with 5 μg/ml hemin and 1 μg/ml menadione (TSBHK) or Bacto™ Brain Heart Infusion Broth (BHI) without sucrose (BD Biosciences) supplemented with 0.5% yeast extract (Fisher Scientific), hemin, and menadione (BHIYHK), or TSBHK supplemented with 5% defibrinated sheep blood (BAPHK) were used for cultivation of *P. gingivalis*. Bacto™ Agar was used for solid media. BHI medium was used for the cultivation of *S. gordonii*. For chemically defined media (CDM), bovine serum albumin (BSA; Alfa Aesar), human serum albumin (HSA, Alfa Aesar), or normal human serum (Merck; Millipore) were dissolved in basal salts (BS) buffer (14 mM Na_2_HPO_4_, 10 mM KCl, 10 mM MgCl_2_, pH 7.3), then filtered (0.22 µm) and supplemented with hemin (H)/menadione (K). For pyruvate-relevant assays, CDM was supplemented with 0.5% pyruvate (Sigma). *P. gingivalis* or *S. gordonii* were incubated at 37 °C anaerobically (5% hydrogen, 10% carbon dioxide, and 85% nitrogen). When necessary, 10% human serum (Merck; Millipore) or 10% saliva in water were prepared and then filter-sterilized (0.22-µm). Saliva from healthy individuals was collected 2 h after food consumption. After chewing a walnut-size ball of sterile parafilm for 10 min, saliva was expectorated into a container on ice until approximately 10 ml of saliva was collected. Following collection, dithiothreitol (DTT) was added to a final concentration of 2.5 mM. Samples were incubated on ice for 10 min followed by removal of particles and debris using centrifugation at 13,000 × *g* for 10 min. Clarified saliva was diluted to 10% with distilled water and then filter-sterilized (0.22-µm). Aliquots of saliva were stored at −20 °C until needed^82^. Enzymes for genetic manipulations and cloning were purchased from New England BioLabs, Ipswich, MA, USA, and all chemicals were purchased from Sigma-Aldrich unless otherwise indicated. Solvents used for the analysis of nucleotide second messengers were HPLC grade.

### Construction of mutants and complementation

Deletions of *pde*_*pg*_ (PGN_0521) and *cdaR* (PGN_1486) were generated through homologous recombination using linear DNA fragments^17,83^. Linear fragments carrying 1-kb flanking regions of the target genes, as well as a promoterless erythromycin resistance gene (*ermF*), were obtained from *P. gingivalis* strain 381 genome and plasmid pVA2198 as a template, respectively, using primers listed in Supplementary Table 2. The PCR was performed using Phusion Hot Start Flex 2X Master Mix (New England BioLabs; cat# M0536S) and consisted of 25 cycles with a temperature profile of 30 s at 98 °C, 30 seconds at 57 °C for annealing, and 10 min at 72 °C for the extension. The NEBuilder HiFi DNA assembly cloning kit (New England BioLabs; cat# E5520S) was used as described in the manufacturer’s protocol for assembling DNA fragments into linear cassettes consisting of the 1 kb upstream region of the gene-*ermF*-1-kb downstream region of the gene. Competent cells were prepared using 15 ml of the actively growing culture of *P. gingivalis* strain 381 (OD_600_ = 0.7). Cells were pelleted and washed twice with electroporation buffer (10% glycerol and 1 mM MgCl_2_; filter-sterilized; stored at 4 °C). The pellet was resuspended in 0.5 ml of electroporation buffer and distributed into 100-μl aliquots. Electroporation was performed using a 100-μl sample of cells to which ~2 μg of the assembled DNA fragments was added and placed in a sterile electrode cuvette (0.2-cm gap). The cells were pulsed with a Bio-Rad gene pulser at 2.5 kV for 5.5 ms and then incubated on ice for 5 min. The cell suspension was then added to 1 ml of TSBHK and incubated for ~16 h. Transformants were selected on BHIYHK plates supplemented with antibiotic followed by further confirmation via genomic DNA isolation using a Wizard^®^ Genomic DNA Purification Kit (Promega) and amplification of upstream/downstream regions of the deleted gene followed by DNA sequencing (Eurofins Genomics)^84^. For in *cis* complementation, the abovementioned strategy for the generation of linear fragments was employed but the linear fragment contained 1-kb flanking regions of the genes, the relevant gene under its native promoter, and the tetracycline resistance gene (*tetQ*) obtained from plasmid pT-COW^85^. Confirmation of gene insertion was by colony selection on antibiotic plates, genome isolation, and amplification of the gene.

### Growth rate analysis and biofilm assays

For growth rate analysis, *P. gingivalis* strains were grown anaerobically on BAPHK for 2–3 days and inoculated in TSBHK or BHIYHK, then incubated for 18 h anaerobically. Cultures grown overnight were centrifuged and the pellets were washed with pre-reduced BS buffer and then suspended in pre-reduced working medium. Bacterial growth was then monitored by measuring the optical density at 600 nm (OD_600_). To estimate the number of viable bacteria, planktonic growth cultures were subsequently prepared at dilutions for plating on BAPHK plates and inoculated anaerobically for 4–5 days. The numbers of colony-forming unit (CFU) of growth culture at different time points were determined.

Biofilm assays were performed in 96-well flat-bottom plates (FALCON^®^, Corning Inc., USA)^16^. To this end, 4 ml of overnight culture was pelleted inside an anaerobic chamber using a mini centrifuge and washed with pre-reduced BS buffer and pelleted. Bacterial cells were resuspended in the working medium supplemented with 5 μg/ml hemin and 1 μg/ml menadione to reach an optical density at OD_600_ = 0.1 (~5 × 10^7^ cells/ml). A total volume of 200 μl of the cells was applied in each well and anaerobically incubated for 48 h. The plates were washed twice by immersing in distilled water to remove free cells, and air-dried. A total volume of 200 μl of 0.1% safranin in water was used to stain each well for 30 min. The plates were washed by immersing twice in distilled water, and air-dried. The extraction of the stain from biofilms was performed using 200 μl of 90% ethanol containing 1% sodium dodecyl sulfate for 30 min followed by reading the absorbance at 492 nm.

For biofilm analysis by confocal laser scanning microscopy, the inoculum was prepared as described above but diluted to OD_600_ = 0.2 and 1 ml of the cells was added to each well of a 12‐well plate (Greiner Bio‐one) containing a circular glass coverslip^16^. Plates were incubated anaerobically for 24 h. Glass coverslips were gently washed with distilled water to remove free cells and stained with either the Invitrogen Live/Dead BacLight Bacterial Viability Kit (Invitrogen) or ViaGram^TM^ Red^+^ Bacterial Gram Stain and Viability Kit (Molecular Probes, Invitrogen) as described by the manufacturer. Stained coverslips were gently placed face down on a glass microscope slide, and biofilms were visualized on a Leica SP8 confocal microscope (Leica Microsystems Inc.), using 507/545 nm (SYTO 9), 614/745 nm (PI), 410/547 nm (DAPI), and 590/750 nm (Texas Red) spectra, and ×63 objective. Images were analyzed using IMARIS image analysis software (Bitplane AG).

### Analysis of nucleotide second messengers

High-performance liquid chromatography coupled with tandem mass spectrometry (HPLC-MS/MS) was employed to perform a comprehensive analysis on various nucleotide second messengers and relevant derivatives^86^. Various internal standards, including ^13^C_20_^15^N_10_-c-di-GMP and ^13^C_20_^15^N_10_-c-di-AMP, were used in this analysis. To this end, biofilm or planktonic cells were prepared as described above. Cells were incubated on ice for 20 min and then harvested by centrifugation at 4700 × *g*, 4 °C for 10 min. Washed cells were resuspended with 300 μL ice-cold extraction solvent containing acetonitrile/methanol/water (2/2/1, v/v/v) and incubated on ice for 15 min. Suspensions were heated at 95 °C for 10 min and cooled again on ice. Following centrifugation at 20,800 × *g* and 4 °C for 10 min, supernatants were collected, and pellets were subjected to extraction twice with solvent without heating. Combined supernatants of three extraction steps were stored at −20 °C overnight for further protein precipitation followed by centrifugation for protein precipitant separation. All residual pellets were collected for protein quantification using the Bicinchoninic Acid (BCA) Kit (Thermo Scientific) and supernatants were dried in a Speed-Vac (Thermo Scientific) at 40 °C. Dried samples were dissolved in 200 μL sample solvent and vortexed for 30 s. After centrifugation, 40 μL of sample solvent with internal standards were directly added in each MS vial with an insert and 40 μL of supernatant were transferred to a MS vial with insert and mixed three times by pipetting. Analysis was performed using liquid chromatography-mass spectrometry (LC-MS).

### Detection and quantification of cNMPs and c-di-NMPs

For the determination of cNMPs, the resuspended samples were mixed 1:2 with an aqueous internal standard solution (100 mg/mL tenofovir). This solution was transferred to measurement vials and analyzed by liquid chromatography (LC) coupled tandem mass spectrometry (5500QTRAP; ABSciex, Framingham, Massachusetts)^87,88^. Cyclic nucleotide monophosphates were separated by reversed-phase chromatography on a C18 column (Zorbax eclipse XCB-C18 50 mm × 4.6 mm; 1.8 µm; Agilent, Santa Clara, California, USA). Solvent A was methanol–water (3:97, v/v), solvent B was methanol–water (97:3, v/v), with each containing 50 mM ammonium acetate and 0.1 vol.% acetic acid. A gradient was used that started with 0% B. Within 5 min, the content of solvent B was increased to 50%. During this time, the elution of the cNMPs was completed. Re-equilibration to the starting conditions was achieved within 3 min. The total run time of the analysis was 8 min at a flow rate of 500 µL/min. Mass spectrometric analysis was performed on a tandem mass spectrometer (QTRAP; MA, USA) using selected reaction monitoring (SRM) in positive ionization mode.

Chromatographic separation of c-di-NMPs was performed by reversed-phase chromatography (RP chromatography) on a C18 column (Nucleodur Pyramid C18 3 µ 50 × 3 mm; Macherey-Nagel; Germany), using water containing 10 mM NH_4_Ac and 0.1% HAc as eluent A, and pure methanol as eluent B. The following gradient was applied: 0 to 4 min, 0% B, 4 to 7.3 min 0–10% B, 7.3–8.3 min 10–30% B and 11–13 min 0% B. The flow rate was set at 600 µL/min. Mass spectrometric analysis was performed on a tandem mass spectrometer (API4000; MA, USA) performing selected reaction monitoring (SRM)^89^. The mass spectrometer was equipped with an electrospray ionization source (ESI) and ionization was performed in positive mode for all analytes.

### ELISA quantification of c-di-AMP and ATP

C-di-AMP was also analyzed using a c-di-AMP Enzyme-Linked Immunosorbent Assay (ELISA) Kit (Cayman Chemical, USA). Briefly, the extraction of metabolites was performed as described above for HPLC-MS/MS. Each sample was assayed at a minimum of two dilutions, each in triplicates. A 50 µl sample was added to each relevant well followed by the addition of 50 µl tracer. Within 15 min, 50 µl of the antibody was added. The plate was covered and incubated for 2 h at room temperature on an orbital shaker. After washing the wells five times with the Wash Buffer of the kit, wells were developed with the addition of 175 µl of TMB (3,3’,5,5’-tetramethylbenzidine) Substrate Solution (Sigma-Aldrich, St. Louis, MO, USA). The reaction was stopped upon adding 75 µl of the kit’s HRP Stop Solution and the plate was read at 450 nm. The standard plot was calculated based on %Bound/Maximum Bound (%B/B_0_) versus serial dilutions of c-di-AMP using a four-parameter logistic fit. The %B/B_0_ value for each sample was also calculated and the concentration of c-di-AMP was identified on the standard curve.

ATP quantification assay was performed using ATPlite^TM^ Luminescence Assay System (https://www.perkinelmer.com) on the same samples explained above. Briefly, a 50 µl of each sample was added to microtiter plate wells in triplicate. 50 µl of the kit’s lysis buffer and 60 µl of the kit’s reconstituting buffer were added and mixed on a rotational shaker (700 rpm) for 5 min. 50 µl of the reconstituted enzyme was added to the wells and mixed for 3 min and immediately the luminescence was measured at 578 nm. The ATP concentration for each sample was calculated on the standard curve which was set up in the same plate using the kit’s ATP standard.

### LPS ELISA assay

For LPS ELISAs, autoclaved extracts of cells were used. Actively growing cells (OD_600_ = 0.7) were pelleted, diluted in sterile distilled water, and normalized to an OD_600_ of 0.5. 1.5 ml of the suspension was pelleted, and half the supernatant was removed. The cells were resuspended in the final 0.75 ml and autoclaved at 120 °C for 30 min. Once cooled, the extracts were centrifuged, and the supernatants were stored for ELISA assay^83^. Autoclaved extracts were diluted in 50 mM carbonate/bicarbonate buffer, pH 9.6, and serially diluted in a 96-well plate which was incubated at 4 °C overnight^90^. Plates were washed with PBS containing 0.05% Tween 20. A solution of 5% milk powder in PBS was used to block wells for 1 h at ambient temperature. After repeated washing, wells were incubated for 1 h at 37 °C with 0.2 µg/ml anti-*Porphyromonas gingivalis* LPS monoclonal antibody (Sigma; SAB4200834). Wells were washed and then incubated for 1 h at 37 °C with a goat anti-mouse IgG2b cross-adsorbed secondary-HRP antibody (Invitrogen; Cat# M32407) diluted at 1:2000 in PBS containing 0.1% Tween 20 and 0.1% bovine serum albumin. After a final wash, wells were incubated with TMB until sufficient color appeared. The reaction was stopped with an equal portion of 1 M HCl, and the absorbance was measured at OD_450_. Pure lipopolysaccharide from *P. gingivalis* (InvivoGen; Cat#tlrl-pglps) was used to plot the standard curve.

### The MIC and SDS sensitivity assays

For MIC test and SDS sensitivity assay, *P. gingivalis* strains were grown anaerobically on BAPHK for 2–3 days and inoculated in TSBHK overnight anaerobically. Cultures grown overnight were centrifuged and the pellets were washed with pre-reduced medium and then suspended in pre-reduced working TSBHK medium to reach OD_600_ = 0.35. Various concentrations of amoxicillin or SDS were added to the cultures. Cultures were incubated anaerobically, and their growth rate was monitored for 24 h in the MIC test and for 8 h in the SDS sensitivity assay. The lowest concentration of antibiotic preventing bacterial growth was considered the MIC (the minimal concentration that inhibited growth by ≥95%).

### Transmission electron microscopy (TEM)

Biofilm cells were harvested and washed with ice-cold PBS three times, and fixation solution (0.1 M phosphate buffer, 2% paraformaldehyde, 2% glutaraldehyde in 20 ml of MilliQ water, pH: 7.4) was added without resuspending the bacteria pellet. The pellet was then refrigerated at 4 °C for at least 48 h. The pellet was washed three times in 0.1 M phosphate buffer for 20 s each wash. 1% osmium tetroxide solution was added and allowed to incubate for 2 h at room temperature. After the osmium solution was removed, the pellets were washed four times (10 min each wash) in 0.1 M phosphate buffer, and the pellets held at 4 °C overnight. A Durcopan resin solution was first mixed up using Durcopan A, B, C, and D components. To a special flask, a 50/50 solution of Durcopan resin and 200 Proof EMS-grade ethanol was made and was set aside for the completion of the pellet dehydration step. Samples were then moved to glass Wheaton sample jars (VWR; Cat# 225536). The 0.1 M phosphate buffer was removed from the sample pellets, and 50%, 70%, 95%, and 100% ethanol were sequentially added, swirled, allowed to sit for 15 min, and removed. Treatment with 100% ethanol was repeated for two more times. The pellet pieces were then added to the 50/50 resin/ethanol mixture and placed on a rotator wheel for 1 h. After the mixture was removed, a 3:1 resin/100% ethanol mixture was added to the pellet samples, and then the samples were placed on a rotator wheel for 1 h, then it replaced with pure resin. The samples were then placed in a compression chamber and allowed to sit overnight. The pellet pieces were then placed into plastic TEM sample capsules, filled completely with resin, and placed into a compression chamber for 1 h (this was done to remove any air bubbles remaining in the resin). The samples were removed, and then placed in an oven set at 60 °C for 2 days for polymerization. The capsules (containing the bacteria pellets) were sectioned using a Leica EM UC7 model ultramicrotome at a thickness of 70 nm. The sections generated were placed on copper EM grids for staining. The sample grids were placed on top of drops of 8% aqueous solution of uranyl acetate for 5 min. Each grid was washed via ten dips in three DI H_2_O-filled beakers. The grids were then allowed to dry for 1 h, and then placed on top of drops of 3% stabilized solution of lead citrate for 2 min. The grids were then washed via ten dips in three DI H_2_O beakers, and then allowed to dry for 1 h. The grids were visualized on a Hitachi HT7700 Transmission Electron Microscope.

### Gingipain assay

To assess the activity of arginine and lysine gingipains, cells were grown anaerobically in 10 ml of BHIYHK to mid-exponential phase. Planktonic cultures were pelleted and normalized to an OD_600_: 1.0, and 1 ml of each culture was centrifuged at 15,000 × *g* for 10 min at 4 °C. The supernatants were transferred to separate tubes while the pellets were resuspended in the gingipain assay buffer containing 200 mM Tris, 5 mM CaCl_2_, 150 mM NaCl, and 10 mM l-cysteine, pH 7.6. The cells and supernatants were diluted 1:10 in the gingipain assay buffer and then each sample was assayed at a minimum of two dilutions in the gingipain assay buffer, each in triplicates. The initial absorbance at 405 nm was recorded and the plates were then incubated at 37 °C for 10 min. The reagents N-α-benzoyl-l-arginine-p-nitroanilide (BAPNA) or N-α-acetyl-l-lysine-p-nitroanilide (ALPNA) was added to the relevant wells at a final concentration of 1 mM and the plates were incubated for 2 h at 37 °C^49^. The final absorbance (405 nm) of the wells was measured and the difference between the initial and final absorbance was determined.

### *Galleria mellonella* infection model

Larvae of *G. mellonella* were used to assess virulence of *P. gingivalis* strain 381 and c-di-AMP relevant mutants as described previously^51,91^. To this end, BHIYHK-grown bacteria were transferred to fresh medium to OD_600_: 0.3 and incubated anaerobically to mid-exponential phase. Bacteria were centrifuged and washed with PBS. The pellets were resuspended in PBS and then normalized to OD_600_: 1.0. Groups of 15 larvae with optimal weight, ranging from 200 to 300 mg, were injected with 5 μl of bacterial inoculum containing ~8 × 10^8^ CFU using a Hamilton syringe. Injection was carried out into the hemocoel of each larva via the last left proleg. After injection, larvae were kept at 37 °C, and survival was recorded at select intervals for up to 160 h.

### Statistical analysis

All experiments were conducted using at least three biological replicates. GraphPad Prism version 9.2.0. was applied for performing Student’s *t* test in pairwise comparisons. For other data, the Shapiro–Wilk test was used to evaluate the normality of the distribution of the data. Parametric data were analyzed by ANOVA followed by post hoc Tukey’s honestly significant difference (HSD) test for pairwise comparisons, using GraphPad Prism version 9.2.0. Differences in the data were considered significant when the probability value was < 5.0% (*P* value < 0.05). The results of virulence assessment using *Galleria mellonella* infection model were analyzed with GraphPad Prism 9.2.0. software by using the Mantel–Cox log-rank test. Experiments were performed independently at least two times.

### Reporting summary

Further information on experimental and research design is available in the Nature Research Reporting Summary linked to this article.

## Supplementary information

Supplementary Materials

Reporting Summary

## Data Availability

The authors declare that the data supporting the findings of this study are available within the paper, and its Supplementary Information files or from the corresponding author upon request.
