# Supplementary material for: Atypical cyclic di-AMP signaling is essential for Porphyromonas gingivalis growth and regulation of cell envelope homeostasis and virulence
Source: NPJ Biofilms Microbiomes. 2022 Jul 6;8:53. doi: 10.1038/s41522-022-00316-w (PMC9259658; doi:10.1038/s41522-022-00316-w)
Supplement: Supplementary file 1 — Supplementary Materials [file 41522_2022_316_MOESM1_ESM.pdf]

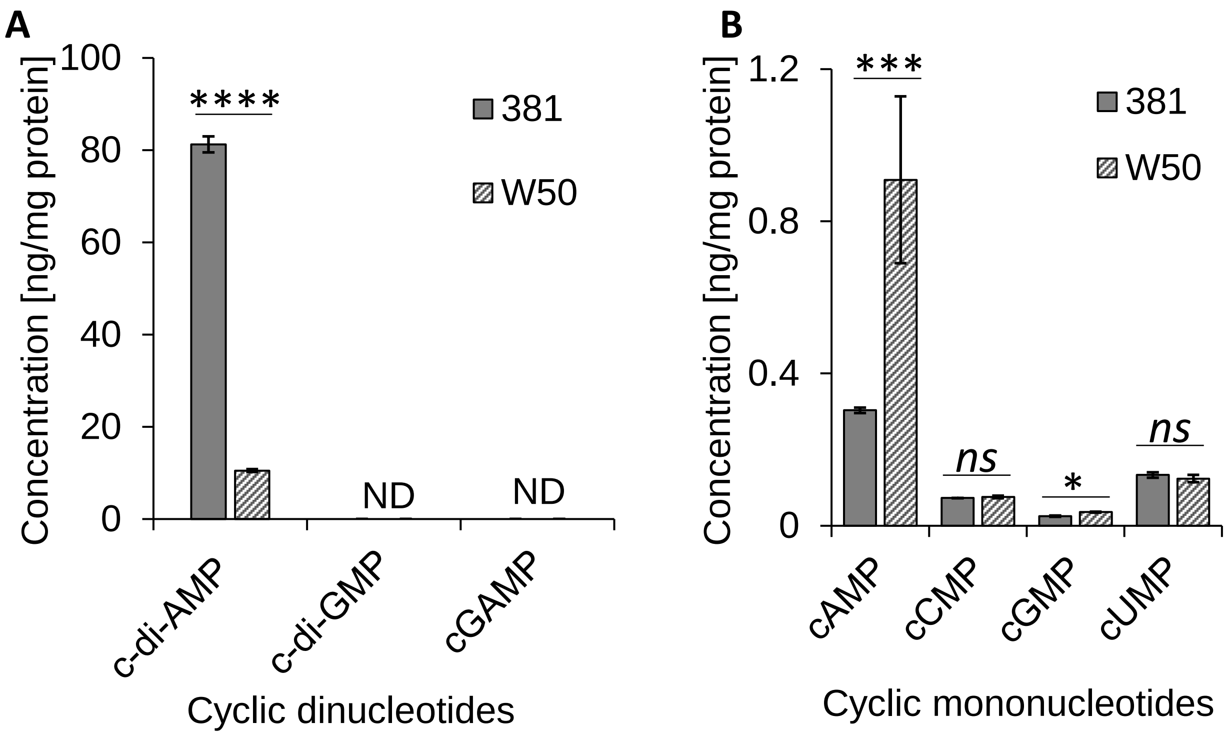

Supplementary figure 1- Comparison of the cellular concentration of cyclic mono- and di-nucleotide second messengers in biofilm cells of *P. gingivalis* strains 381 and W50. A) Graph shows the cellular levels of cyclic dinucleotide molecules, and B) shows the cellular levels of cyclic mononucleotide second messengers. Graphs represent the mean ± SE (three biological replicates) of nucleotide second messengers which were analyzed with a student's *t* test (*$p < 0.05$; *** $p < 0.001$; **** $p < 0.0001$; *ns* not significant). ND, not detected.

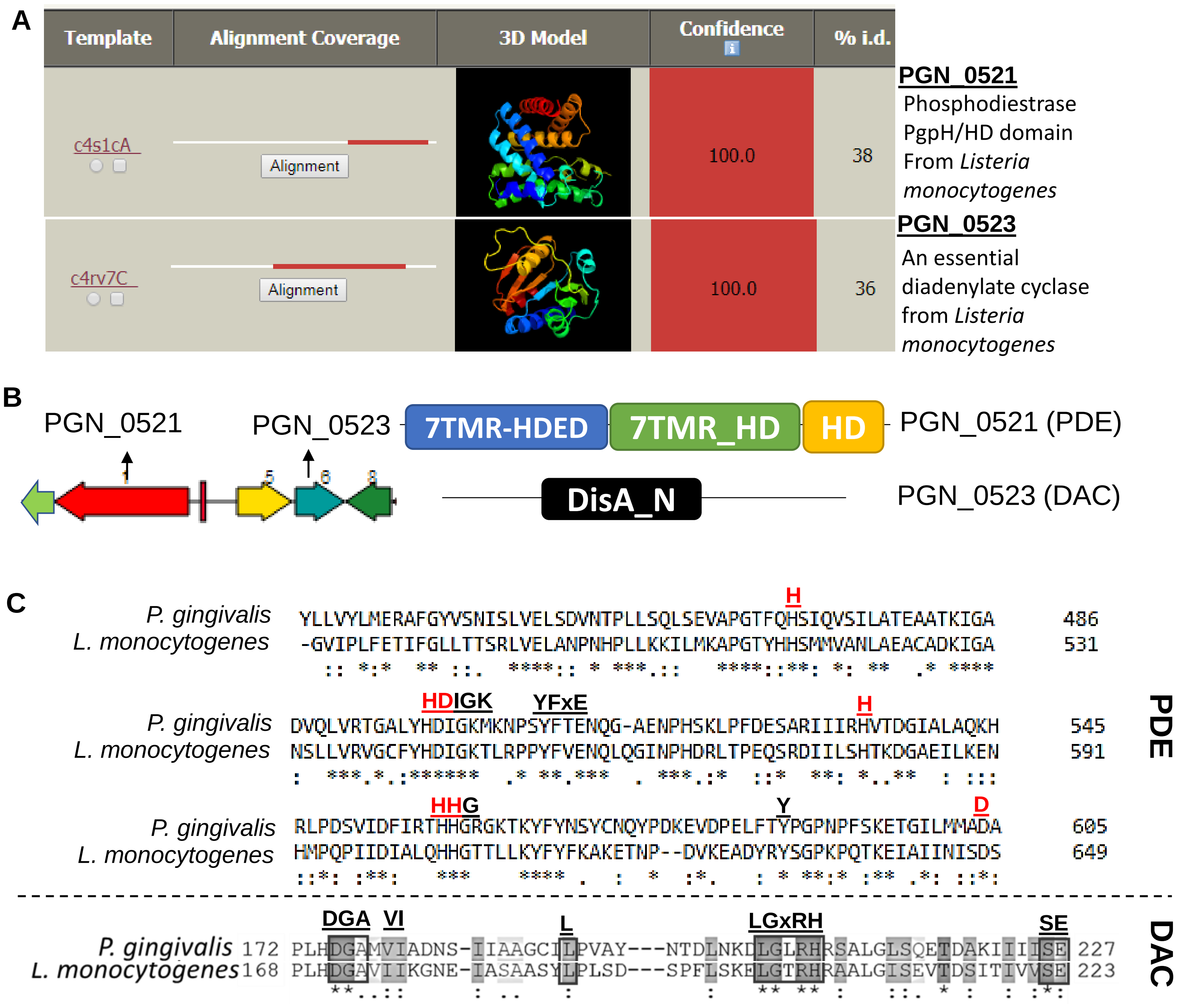

Supplementary figure 2- Bioinformatics analysis of predicted c-di-AMP DAC and PDE enzymes in *P. gingivalis*. A) Structural models developed by the Phyre2 Protein Fold Recognition Server show sequence homology of DAC$_{pg}$ and PDE$_{pg}$ to *Listeria monocytogenes* DAC and PgpH/HD-type PDE enzymes, respectively. B) Proximity of dac_{pg} and pde_{pg} on the genome of *P. gingivalis* strains and predicted conserved domains involved in c-di-AMP synthesis or degradation implemented by the SMART protein domain annotation resource. C) ClustalW2 multiple sequence alignment of DAC$_{pg}$ or PDE$_{pg}$ and *L. monocytogenes* DAC or PgpH/HD-type PDE predicted highly conserved amino acids involved in catalytic activities of DAC$_{pg}$ or PDE$_{pg}$. Red letters are c-di-AMP binding amino acids in *L. monocytogenes* PgpH/HD-type PDE (PDB code: 4S1C).

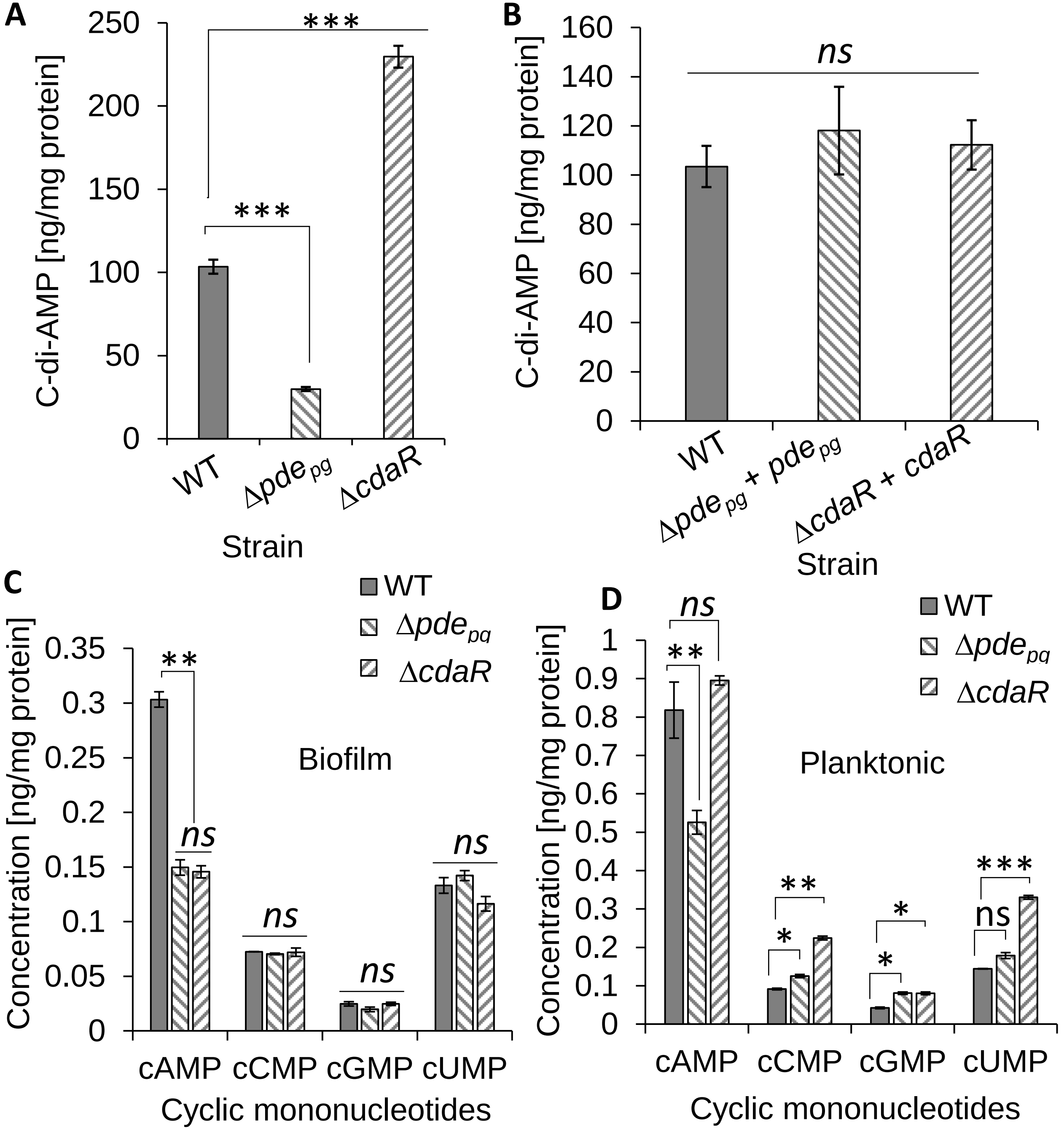

Supplementary figure 3- Comparison of the cellular concentration of c-di-AMP, pApA, and cyclic mononucleotides in biofilm cells of *P. gingivalis* strains 381, Δ*pde*$_{pg,}$ and Δ*cdaR* mutants or complemented strains. A) Graph shows the cellular levels of c-di-AMP in the wild type and mutants, and B) shows the cellular levels of c-di-AMP in *cis*-complemented mutants with relevant genes. C and D) cellular concentrations of cyclic mononucleotides in the mutants and the wild type. E and F) Graphs show respectively the cellular levels of pApA and AMP in the wild type and mutants. Graphs represent the mean ± SE (three biological replicates) of quantified molecules which were analyzed with a student's *t* test (*$p < 0.05$; **$p < 0.01$; *** p <0.001; **** p <0.0001; *ns* not significant).

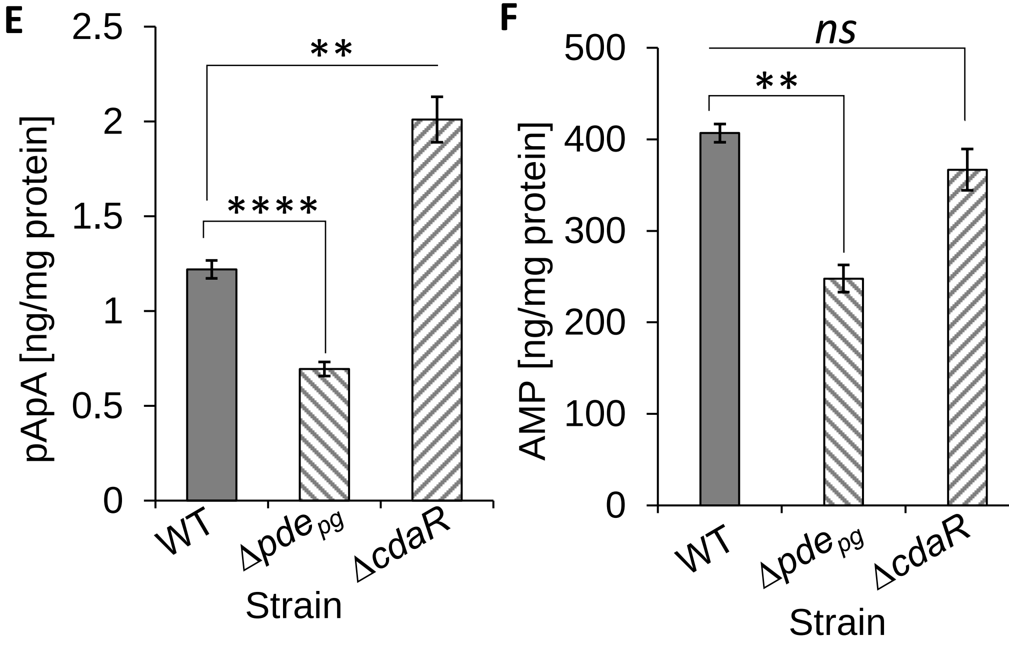

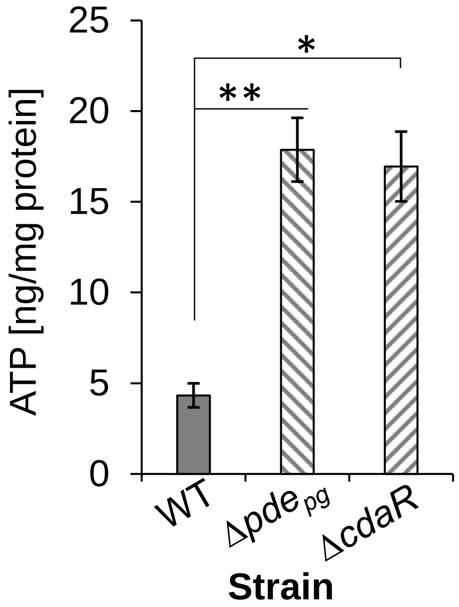

Supplementary figure 4- Comparison of the cellular concentration of ATP in biofilm cells of *P. gingivalis* strains 381, Δ*pde*$_{pg,}$ and Δ*cdaR* mutants. Graph represents the mean ± SE (three biological replicates) of quantified ATP which were analyzed with a student's *t* test (*$p < 0.05$; **$p < 0.01$).

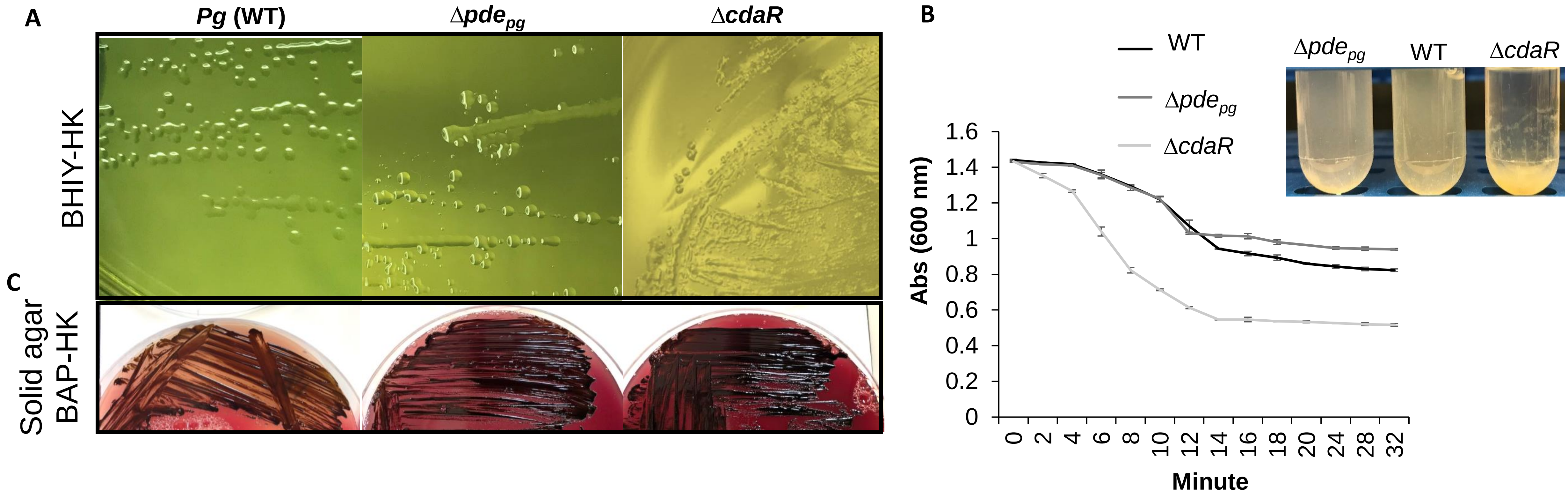

Supplementary figure 5- Comparison of colony morphology and the growth rate of the wild type, Δ*pde*$_{pg}$, and Δ*cdaR* mutants. A) Strain 381 displays a smooth appearance on BHIY-HK plates but the colony of Δ*pde*$_{pg}$ has a glossier appearance while that of Δ*cdaR* appeared as a rough and cohesive colony. B) Autoaggregation assay in the CDM-BSA-HK medium demonstrates the strong cohesiveness and auto-aggregation of Δ*cdaR* cells and less auto-aggregation ability of Δ*pde*$_{pg}$ cells compared to the parent strain 381. C) WT and both mutants form black-pigmented colonies on blood agar plates after a week of anaerobic incubation. After two weeks, only the wild type showed a strong clear zone around the colonies due to strong hemolytic activity while the mutants did not.

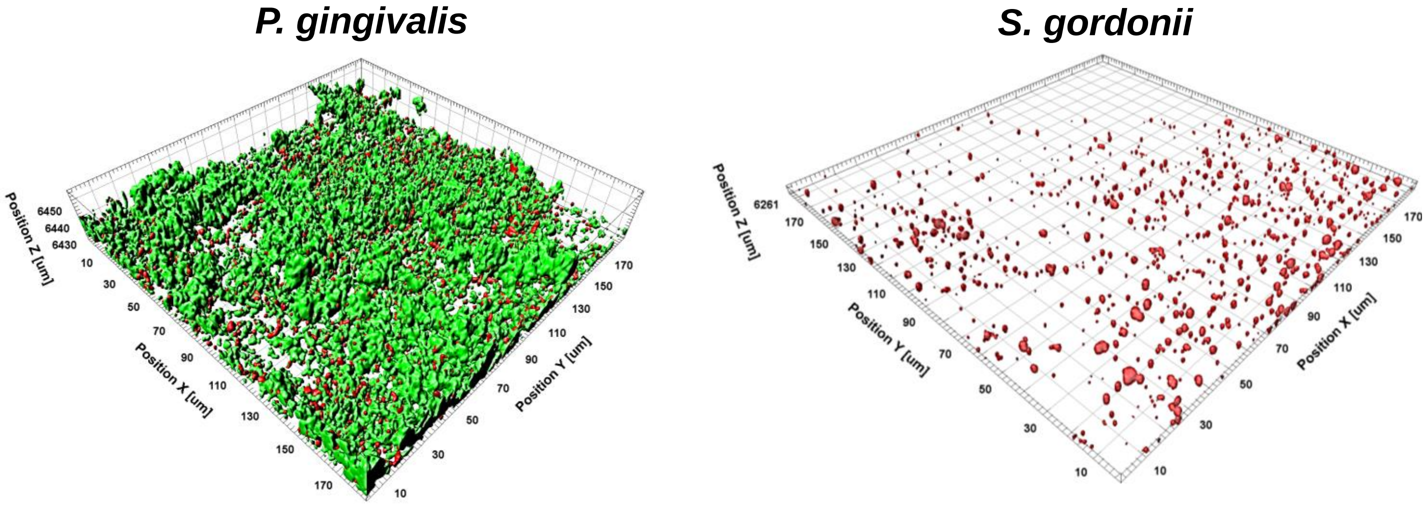

Supplementary figure 6- The CLSM images of mono-species biofilms of *P. gingivalis* or *S. gordonii* in the CDM-Serum-HK medium (Calculated biofilm parameters are presented in Fig. 3).

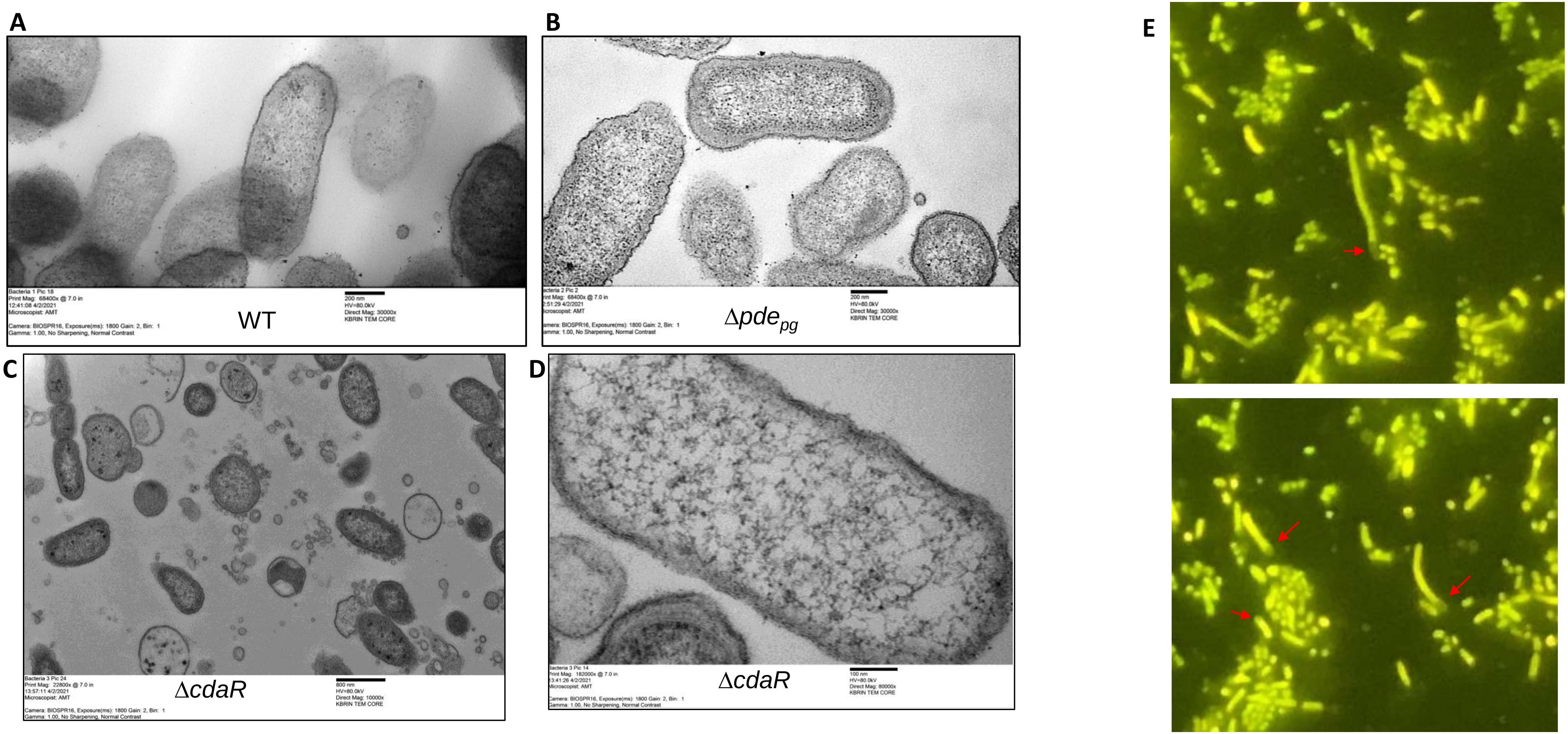

Supplementary figure 7- TEM images of strain 381 and Δ*pde*$_{pg}$ and Δ*cdaR* mutants. A-D) Images show differences in cell morphology and cell envelope structure between the wild type and mutants. E) Consistent with TEM images (C), CLSM images represent Δ*cdaR* cells with void structures in their cell envelope as a result of dysregulation of membrane homeostasis (red arrows).

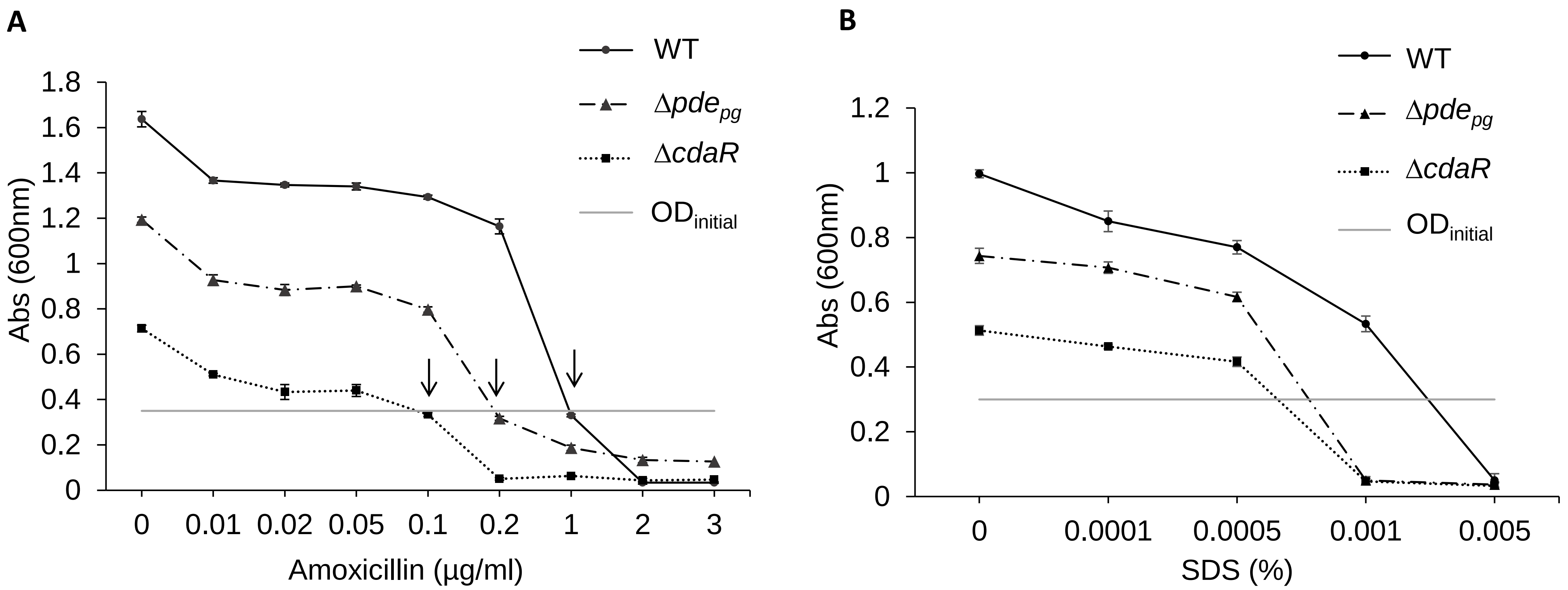

Supplementary figure 8- Comparison of the susceptibility of strain 381, Δpde_{pg}, and Δ*cdaR* mutants to amoxicillin and sodium dodecyl sulfate (SDS). A) The MIC test shows that growth of the mutants was inhibited at lower MIC of amoxicillin compared to the parent strain 381. Arrows indicate amoxicillin concentrations at which growth of strains was inhibited within 24 hours. B) Graph shows that, after 8 hours, Δpde_{pg} and Δ*cdaR* cells were completely lysed in the presence of 0.001% SDS while the wild type did not. Graphs represent the mean ± SE (three biological replicates) of the MIC test and SDS sensitivity assay in static TSBHK liquid cultures.

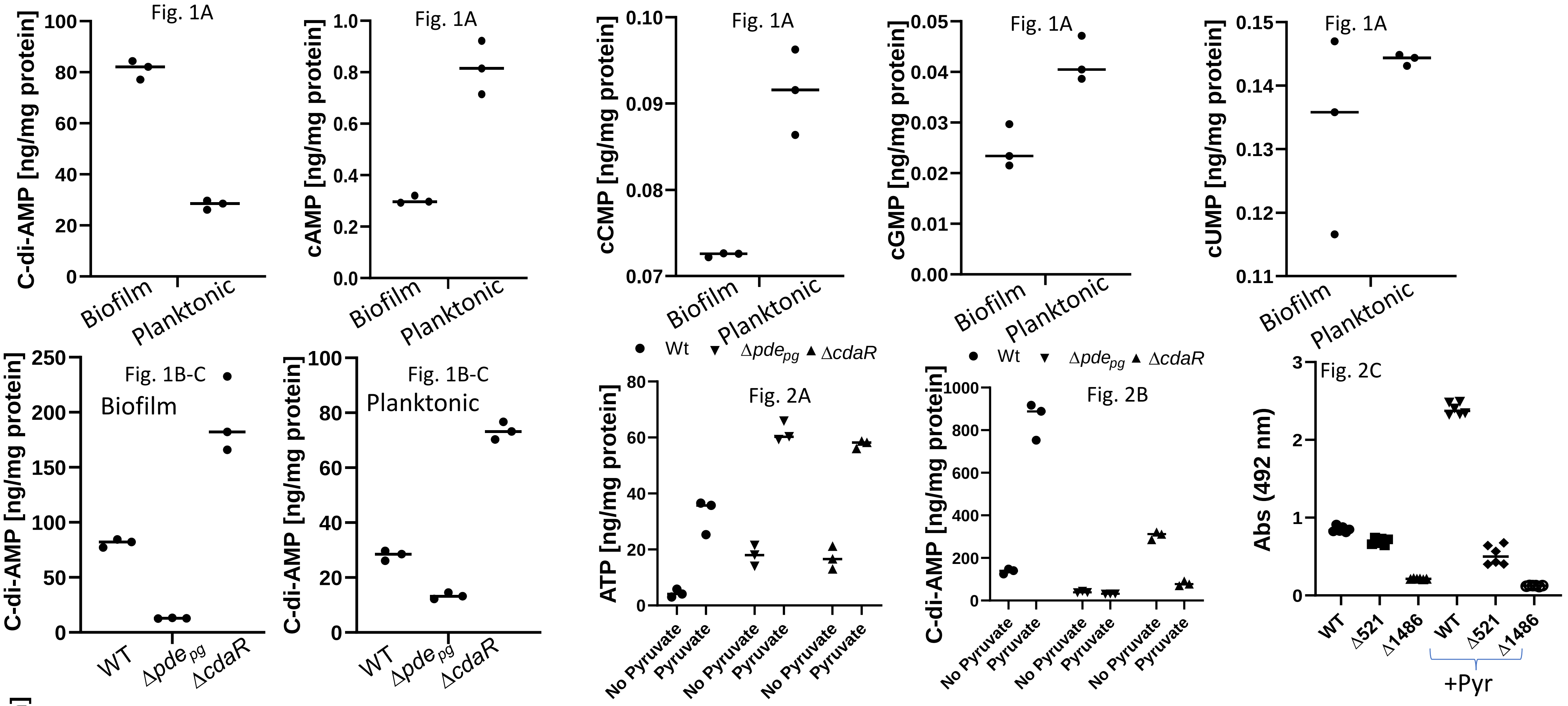

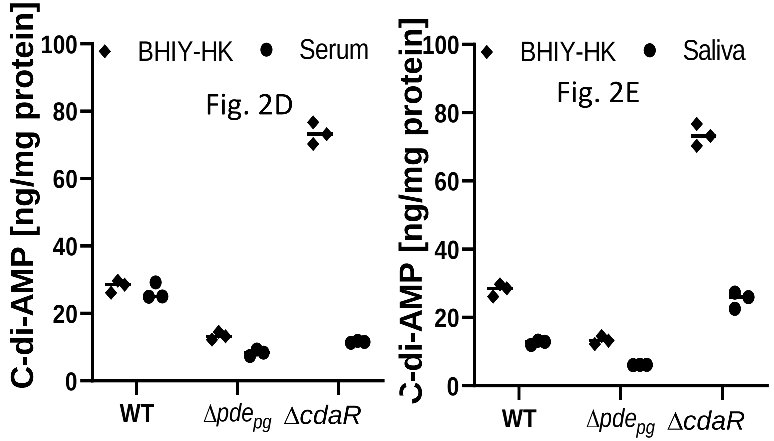

Supplementary figure 9- Scatter plots displaying the data distribution presented in each figure.

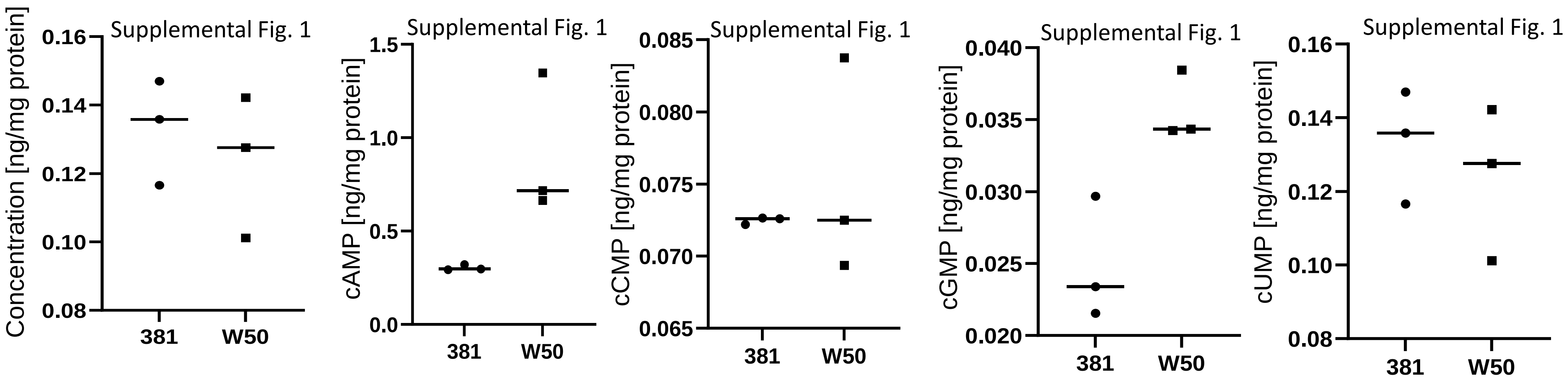

Supplementary figure 9- Scatter plots displaying the data distribution presented in each figure.

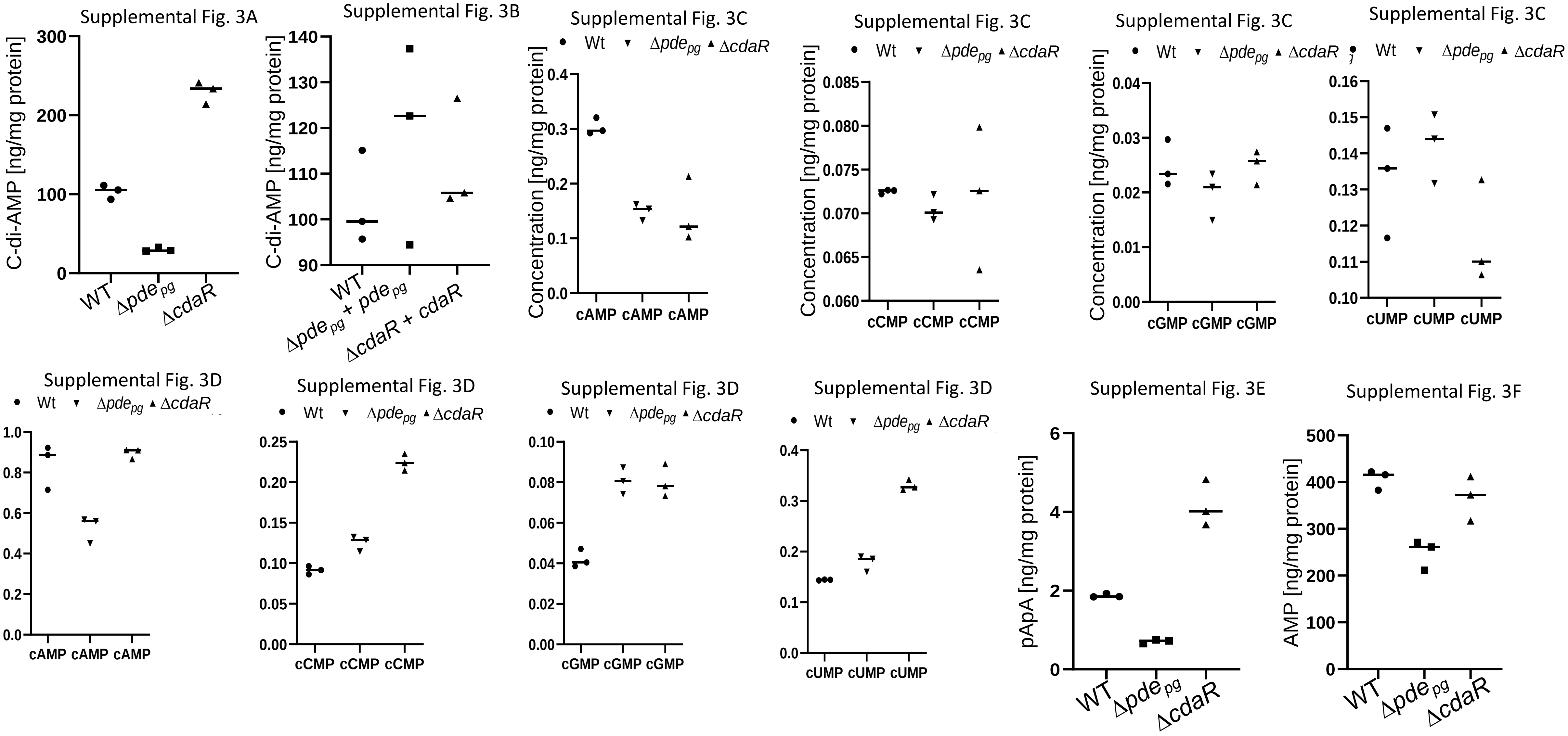

Supplementary figure 9- Scatter plots displaying the data distribution presented in each figure.

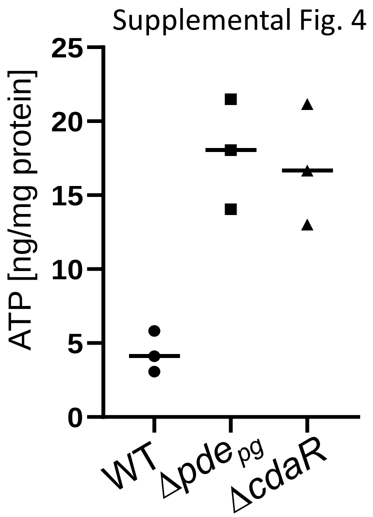

Supplementary figure 9- Scatter plots displaying the data distribution presented in each figure.

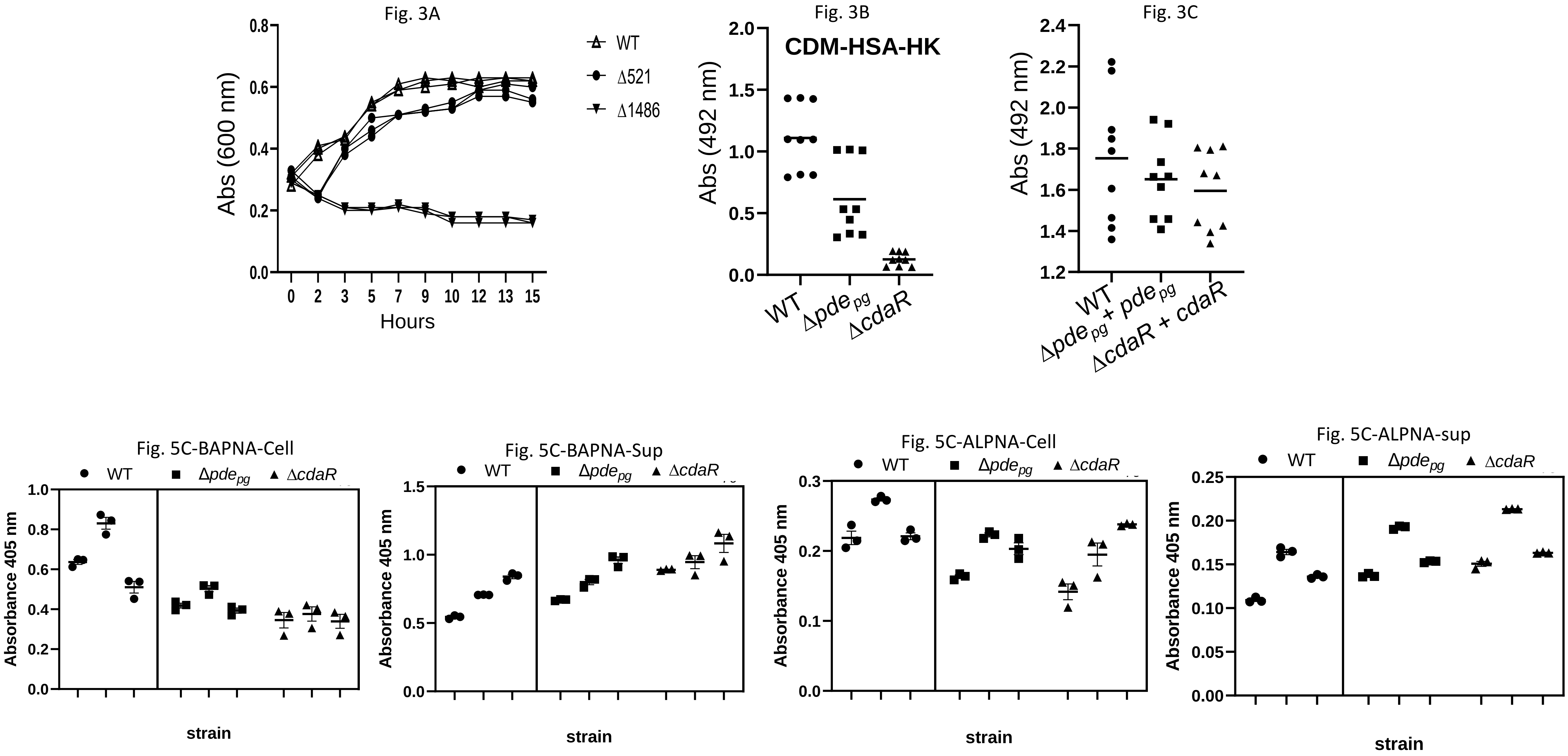

Supplementary figure 9- Scatter plots displaying the data distribution presented in each figure.

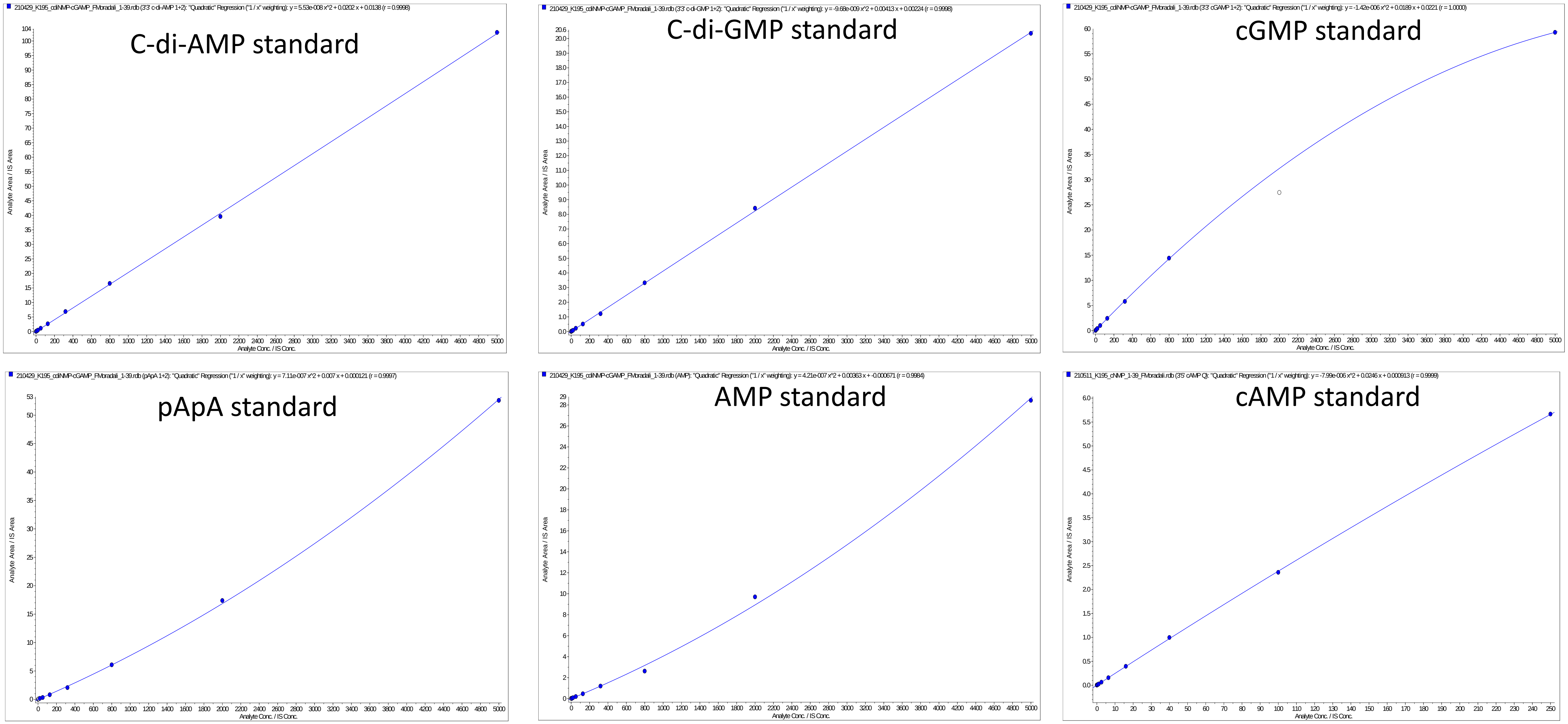

Supplementary figure 10- Graphs show standard curves which are used in this study.

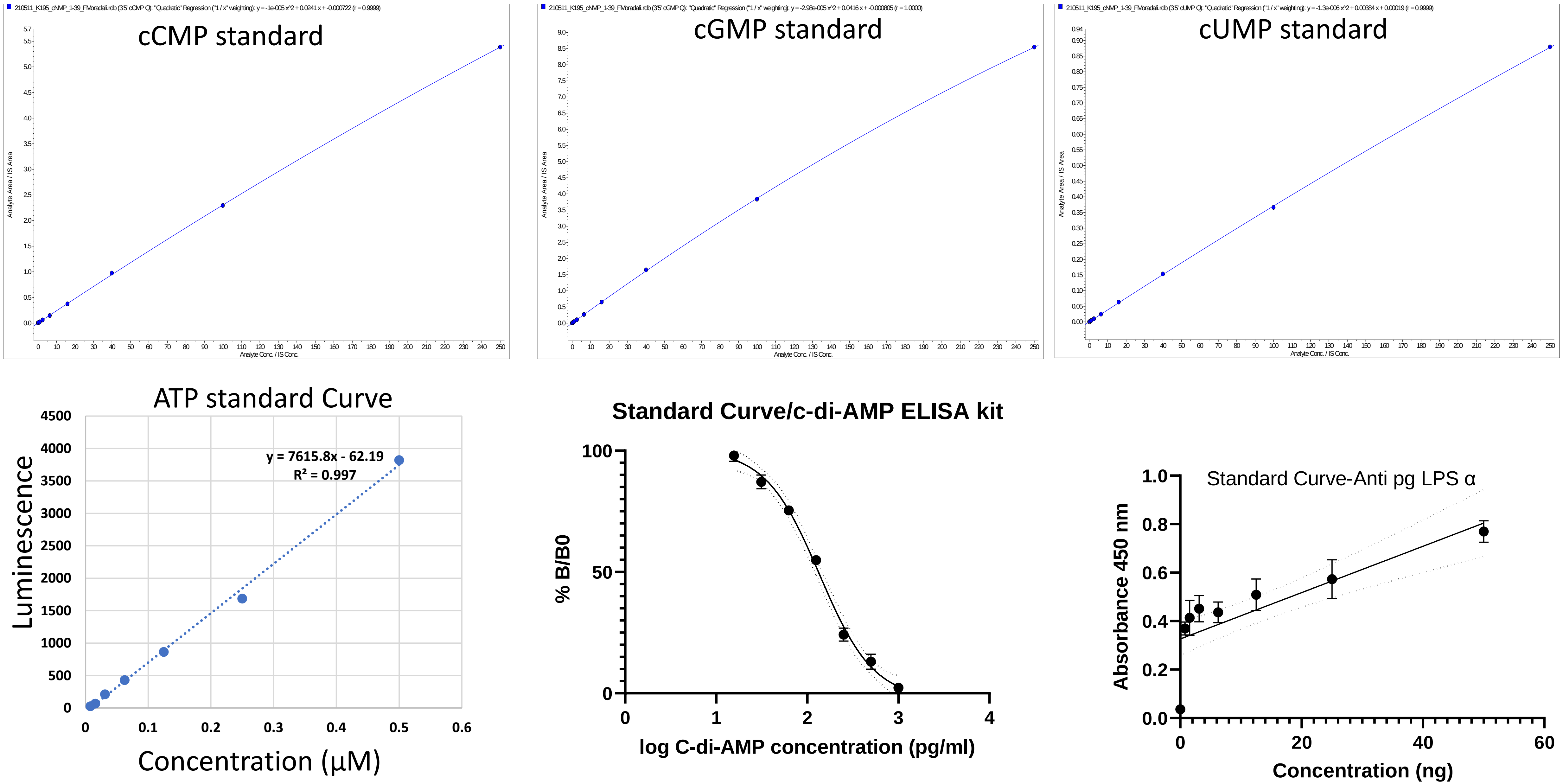

Supplementary figure 10- Graphs show standard curves which are used in this study.

**Supplementary Table 1**							
**The mean values of cNMPs, c-di-NMPs, and other molecules expressed in pmol/mg protein**							
**Strain/mutant**	**c-di-AMP**	**cAMP**	**cCMP**	**cGMP**	**cUMP**	**pApA**	**AMP**
Strain 381 (Biofilm)	123.4 ± 2.65	0.92 ± 0.02	0.24 ± 0.0003	0.072 ± 0.006	0.43 ± 0.024	2.78 ± 0.03	1171.6 ± 28.5
Strain 381 (Planktonic)	42.71 ± 1.3	2.49 ± 0.22	0.3 ± 0.008	0.122 ± 0.007	0.47 ± 0.001	1.8 ± 0.07	931.6 ± 76
Strain W50 (Biofilm)	16 ± 0.47	2.76 ± 0.66	0.25 ± 0.01	0.1 ± 0.003	0.4 ± 0.03	0.72 ± 0.07	1570.1 ± 53.4
Δpde_{pg} (Biofilm)	19.56 ± 0.18	0.45 ± 0.02	0.23 ± 0.002	0.057 ± 0.006	0.46 ± 0.015	1.05 ± 0.035	713.9 ± 43.3
Δpde_{pg} (Planktonic)	20.28 ± 0.84	1.6 ± 0.09	0.41 ± 0.014	0.23 ± 0.008	0.58 ± 0.025	1.03 ± 0.05	1280.5 ± 40.3
Δ*cdaR* (Biofilm)	294 ± 25	0.44 ± 0.01	0.23 ± 0.012	0.07 ± 0.004	0.38 ± 0.022	6.2 ± 0.41	1056.5 ± 64.6
Δ*cdaR* (Planktonic)	111.5 ± 2.3	2.72 ± 0.03	0.73 ± 0.015	0.23 ± 0.01	1.07 ± 0.016	2.98 ± 0.18	1438.7 ± 16.5
**In response to human serum**							
Strain 381 (Planktonic)	40.1 ± 1.7	1.7 ± 0.21	0.24 ± 0.004	0.16 ± 0.007	0.36 ± 0.015	1.89 ± 0.017	964.6 ± 26.1
Δpde_{pg} (Planktonic)	13.4 ± 0.4	0.77 ± 0.06	0.4 ± 0.04	0.15 ± 0.005	0.47 ± 0.1	0.66 ± 0.14	899.7 ± 86.4
Δ*cdaR* (Planktonic)	17.5 ± 0.2	0.62 ± 0.2	0.11 ± 0.004	0.04 ± 0.003	0.63 ± 0.25	0.83 ± 0.05	863.4 ± 10.1
**In response to human saliva**							
Strain 381 (Planktonic)	19.2 ± 0.5	0.71 ± 0.05	0.01 ± 0.013	0.04 ± 0.002	0.26 ± 0.05	2.71 ± 0.11	1165.3 ± 34.5
Δpde_{pg} (Planktonic)	9.2 ± 0.05	0.8 ± 0.03	0.12 ± 0.005	0.06 ± 0.004	0.54 ± 0.04	0.78 ± 0.05	1177.5 ± 15.6
Δ*cdaR* (Planktonic)	38.4 ± 1.7	0.31 ± 0.07	0.01 ± 0.01	0.03 ± 0.002	0.84 ± 0.06	2.86 ± 0.17	1285.3 ± 77.8
***Cis*-complementation**							
Δpde_{pg} + pde_{pg} (Biofilm)	179 ± 27.1						
Δ*cdaR* + *cdaR* (Biofilm)	171 ± 15.2						
Strain 381 (Biofilm)	157 ± 12.7						
**Addition of pyruvate**							
	**c-di-AMP**	**ATP**					
Strain 381 (-pyruvate)	209.5 ± 8.5	8.54 ± 1.3					
Strain 381 (+pyruvate)	1295.6 ± 63	64.2 ± 5.9					
Δpde_{pg} (-pyruvate)	60.50 ± 2.5	35.2 ± 3.5					
Δpde_{pg} (+pyruvate)	46.8 ± 0.21	121.9 ± 3.3					
Δ*cdaR* (-pyruvate)	465.1 ± 13.3	33.41 ± 3.8					
Δ*cdaR* (+pyruvate)	120.2 ± 7.8	113.7 ± 1.3					

**Supplementary Table 2 -** Primers used in this study	
**Deletion of *dac*$_{pg}$**	
Upstream *dac*$_{pg}$ (PGN_0523).Fw<br>accggaatgtgcatccaatgggtgt	
Upstream *dac*$_{pg}$ (PGN_0523).Rv<br>tcttttttgtcatGAGATTGAGATGTTTTTTGTGGAGTGAGAATTTC	
Downstream *dac*$_{pg}$ (PGN_0523).Fw<br>tccttcgtagTGCCGAGGAATTTCCTCAGAAGAG	
Downstream *dac*$_{pg}$ (PGN_0523).Rv<br>tagccgctttccgccgaaaagctga	
Erm.Fw<br>tctcaatctcATGACAAAAAAGAAATTGCCCG	
Erm.Rv<br>ttcctcggcaCTACGAAGGATGAAATTTTTCAG	
**Deletion of *pde*$_{pg}$**	
Upstream *pde*$_{pg}$ (PGN_0521).Fw<br>agaataagcaccgacgtcgatcaag	
Upstream *pde*$_{pg}$ (PGN_0521).Rv<br>tcttttttgtcatCTTCATCTTCATTGCGAGAAAGTTAAATTATAG	
Downstream *pde*$_{pg}$ (PGN_0521).Fw<br>tccttcgtagTCGTTTCAGGTGCTTCACTATTG	
Downstream *pde*$_{pg}$ (PGN_0521).Rv<br>ctatgctgcggtagcttctatcctg	
Erm.Fw<br>gaagatgaagATGACAAAAAAGAAATTGCCCG	
Erm.Rv<br>cctgaaacgaCTACGAAGGATGAAATTTTTCAG	
**Deletion of *cdaR***	
Upstream *cdaR* (PGN_1486).Fw<br>atgggcatcagctttgcatgtgcga	
Upstream *cdaR* (PGN_1486).Rv<br>tcttttttgtcatTCCCTTTAGCGGATTTGGTGGAG	
Downstream *cdaR* (PGN_1486).Fw<br>tccttcgtagGCAGCAATGATTACCATTGGGATC	
Downstream *cdaR* (PGN_1486).Rv<br>gaagaaagcacgtagtgagtcgttg	
Erm.Fw<br>gctaaagggaATGACAAAAAAGAAATTGCCCG	
Erm.Rv<br>tcattgctgcCTACGAAGGATGAAATTTTTCAG	
***cis*-complementation of Δ*pde*$_{pg}$**	
1KUp+ORF521_fwd	agaataagcaccgacgtcgatcaag
1KUp+ORF521_rev	tcgttgtttattcagacggctccttgtgtctcttc
TetQ-with Promoter_fwd	gagccgtctgaataaacaacgaattatctccttaacg
TetQ-with Promoter_rev	acctgaaacgattattttgatgacattgatttttggaac

1KDown of 0521_fwd	catcaaaataatcgtttcaggtgcttcactattgg
1KDown of 0521_rev	ctatgctgcggtagcttctatcctg
cis*-complementation of Δ*cdaR	
1KUp1486-FW	atgggcatcagctttgcatgtgcg
1KUp-ORF1486-RV	ttcgttgtttatttactgaatgcgttcgatgatgaattgg
TetQ-with Promoter_fwd	cgcattcagtaaataaacaacgaattatctcc
TetQ-with Promoter_fwd	taatcattgctgcttattttgatgac
1KDown1486-FW	gtcatcaaaataagcagcaatgattaccattgg
1KDown1486-Rv	gaaagcacgtagtgagtcgttgtcc
